# A Meta-Analysis of the Faking Resistance of Forced-Choice Personality Inventories

**DOI:** 10.3389/fpsyg.2021.732241

**Published:** 2021-09-29

**Authors:** Alexandra Martínez, Jesús F. Salgado

**Affiliations:** Department of Political Science and Sociology, Faculty of Labor Relations, University of Santiago de Compostela, Santiago de Compostela, Spain

**Keywords:** faking, Big Five, forced-choice inventories, personnel selection, hiring decisions, meta-analysis

## Abstract

This study presents a comprehensive meta-analysis on the faking resistance of forced-choice (FC) inventories. The results showed that (1) FC inventories show resistance to faking behavior; (2) the magnitude of faking is higher in experimental contexts than in real-life selection processes, suggesting that the effects of faking may be, in part, a laboratory phenomenon; and (3) quasi-ipsative FC inventories are more resistant to faking than the other FC formats. Smaller effect sizes were found for conscientiousness when the quasi-ipsative format was used (δ = 0.49 vs. δ = 1.27 for ipsative formats). Also, the effect sizes were smaller for the applicant samples than for the experimental samples. Finally, the contributions and practical implications of these findings are discussed.

## Introduction

The possibility that individuals may intentionally distort their responses to non-cognitive assessment procedures (e.g., resumes, personality inventories, assessment centers, interviews, and biodata, among others) has been a recurrent issue in work and organizational (W/O) psychology (Aamodt, 2003; Levashina and Campion, 2007; Griffith and Converse, 2012; Delgado-Rodríguez et al., 2018; García-Izquierdo et al., 2020; Golubovich et al., 2020). In the last decades, this intentional distortion has been widely researched due to the increasing use of personality inventories in personnel selection processes (Griffth and Peterson, 2006; Ziegler et al., 2012). This behavior has been given different names, for example, social desirability, response distortion, sincerity, lie, or impression management. Currently, the most widely used label is faking.

Two main viewpoints can be distinguished in the study of faking. The first describes faking as an irrelevant phenomenon whose effects on selection instruments and processes are minimal or non-existent (e.g., Hough et al., 1990; Ellingson et al., 2001; Hogan et al., 2007). The second viewpoint has considered faking as a real problem for non-cognitive procedures, particularly, personality inventories (e.g., Viswesvaran and Ones, 1999; Donovan et al., 2003; Birkeland et al., 2006; Griffith et al., 2007; Salgado, 2016). The current empirical evidence strongly supports the second approach. Individuals can voluntarily fake their answers to inventories, which can negatively affect the whole evaluation process (Salgado, 2016). In addition, faking is a phenomenon that can occur in any organization, regardless of the sector, that uses personality inventories and other non-cognitive procedures open to faking (Griffith and Converse, 2012; García-Izquierdo et al., 2019).

The pervasive effects of faking have increased the interest in finding procedures that can control the negative consequences of this phenomenon (e.g., artificial modification of candidates' ranking). However, the nature of faking has made this a complex task, leading to a variety of assessment procedures with different degrees of effectiveness. Among them, forced-choice (FC) inventories stand out as an instrument capable of reducing the effects of faking.

FC personality inventories are characterized by the presentation of item sets (more frequently, pairs, triads, tetrads) with a similar degree of social desirability (Converse et al., 2006; Dilchert and Ones, 2012; Stark et al., 2014). This makes it more difficult to fake. Primary studies and meta-analyses have shown FC personality inventories' effectiveness in reducing the effects of faking (e.g., Nguyen and McDaniel, 2001; Cao and Drasgow, 2019). However, the meta-analyses that have examined FC personality inventories' robustness against faking have some methodological weaknesses. Therefore, a new meta-analysis that overcomes those weaknesses might be a relevant contribution to the literature. Thus, this study's objectives are two-fold: (a) to meta-analytically examine the resistance of the FC inventories to the effects of faking and (b) to determine the potential moderating influence of the study design and the format of FC inventories on the degree of faking.

### Faking Behavior

Faking is one of the most harmful phenomena in W/O psychology and personnel selection because faking always results in an artifactual modification of the candidates' ranking. Consequently, assessment procedures predict performance less accurately, and hiring decisions contain more errors.

In the literature, faking has been approached from several perspectives. For instance, it has been studied as a personality trait, as a response bias, as a response style, or as a mechanism that reduces the assessment procedures' reliability and validity (Paulhus, 1986, 2002; Zickar et al., 2004; Griffith et al., 2006; Ziegler et al., 2009; Burns and Christiansen, 2011; Salgado, 2016).

However, despite the different points of view, four essential conclusions have been reached (Ziegler et al., 2012, 2015): (1) faking is a behavior, not a personality trait; (2) individuals show a distorted image of themselves through faking; (3) to fake individuals must be motivated by the desire to achieve a specific objective; and (4) individual characteristics and contextual variables affect the intention to fake. Based on these four aspects, faking can be defined as an intentional distortion of the responses to the assessment procedures in order to obtain some benefit or advantage in the assessment processes (Zickar and Gibby, 2006; Levashina and Campion, 2007; Ziegler et al., 2012; Salgado, 2016).

Although various faking taxonomies exist, two faking types are generally distinguished: faking good and faking bad (e.g., Sackett et al., 1989; Levin and Zickar, 2002; Zickar et al., 2004; Levashina and Campion, 2007; Kim, 2011). On the one hand, faking good happens when individuals try to show a better image of themselves to obtain better scores in some of the variables or procedures used in the assessment process. This behavior is mainly related to personnel selection processes and academic decisions. On the other hand, faking bad happens when individuals try to show a more negative image of themselves in order to obtain worse scores in the assessment process. Faking bad is more frequent in forensic and clinical contexts where individuals believe that the simulation of disorders will support them, for instance, in a legal process (Salgado, 2005, 2016). In the present research, we have focused on faking good because it is the type of faking that would occur in hiring decision processes (e.g., Rosse et al., 1998; Donovan et al., 2014).

### Effects of Faking

Even though faking can affect all sorts of non-cognitive procedures, the studies of the effects of faking have largely focused on personality inventories rather than other assessment procedures, partially because they are one of the most extensively used instruments in high-stakes decision processes (Rothstein and Goffin, 2006), and, partially, because several meta-analyses have shown that personality factors are relevant predictors of both occupational and academic performance (e.g., Salgado, 2003, 2017; Judge et al., 2013; Salgado and Táuriz, 2014; Salgado et al., 2015; to mention some of the most recent). However, one of the main criticisms of personality inventories is their susceptibility to faking (Rosse et al., 1998; Viswesvaran and Ones, 1999; McFarland and Ryan, 2000; Christiansen et al., 2005; Griffith and McDaniel, 2006; Morgeson et al., 2007a,b; Martínez, 2019). So, it is understandable that the research has focused on finding out the real effects of faking on these instruments (Salgado, 2016).

Previous research has shown that faking has significant negative consequences on the psychometric properties of personality inventories. The main effects of faking are an increase in the mean and a decrease in the standard deviation (*SD*) of the distribution of personality variables. In addition, empirical evidence has shown that faking also produces a decrease in reliability and in criterion-oriented validity and modifies the inventory's factor structure. These effects have been found in both primary studies and meta-analyses (e.g., Hough et al., 1990; Douglas et al., 1996; Viswesvaran and Ones, 1999; Birkeland et al., 2006; Hooper, 2007; Salgado, 2016; Salgado and Lado, 2018).

For instance, the meta-analysis of Viswesvaran and Ones (1999) found that, in experimental settings, the effect size under faking conditions was *d* = 0.50 in the between-subject designs, while for the within-subject designs, it was *d* = 0.70. Therefore, they concluded that (a) faking increases the scores on personality dimensions and (b) this effect is larger for the within-subject designs.

The meta-analyses of Birkeland et al. (2006), Hooper (2007), and Salgado (2016) also found that faking increases the scores in occupational settings. These meta-analyses compared actual job applicant samples and non-applicant samples (i.e., incumbents or respondents in a non-applicant context). The results showed that job applicants scored higher than non-applicants, particularly on conscientiousness and emotional stability. Birkeland et al. (2006) found an average *d* = 0.30 in actual applicant samples, and the average effect sizes found by Hooper (2007) and Salgado (2016) were 0.53 and 0.70, respectively. In addition, Salgado (2016) also found that faking reduced the magnitude of the standard deviations and of the reliability coefficients. Hence, meta-analytic evidence showed that, in personnel selection, faking distorts the scores of the personality factors: faking produces an artificial increase in the mean of the scores and reduces the standard deviations.

The faking effects found in those meta-analyses can have a negative impact on the hiring-decisions in selection processes as they could produce significant changes in the selection ranking of candidates. In other words, applicants who fake their personality inventories would be undeservedly in higher ranking positions than those applicants who have not faked. Consequently, practitioners would make wrong hiring decisions during assessment processes based on those personality inventory answers (Griffith et al., 2007).

It is important to remark that those meta-analyses were conducted with primary studies that mainly used single-stimulus personality inventories (e.g., NEO-PI-R, 16PF, MMPI, CPI, and similar), and that the number of studies that used FC personality inventories was marginal. Therefore, it might be said that, until very recently, faking effects were tested for the SS personality inventories only. This suggests that a closer examination of the characteristics of the FC personality inventories and the empirical evidence of their robustness against faking is required.

### Forced-Choice Inventories

The susceptibility of SS inventories to the effects of faking has led to the search for other mechanisms to reduce the impact of faking on non-cognitive assessment procedures in general and on personality inventories in particular. In this sense, FC inventories are one type of assessment procedure which has been suggested as a means of controlling and reducing the effects of faking (when compared with SS personality inventories). Original research about FC personality inventories dates back to the 1940s, but it was not until recently that there has been significant interest from both researchers and practitioners. The interest in FC personality inventories is due to the evidence of their validity for predicting job performance and training proficiency (e.g., Salgado and Táuriz, 2014; Salgado et al., 2015; Salgado and Lado, 2018), as well as their potential robustness against faking (Nguyen and McDaniel, 2001; Cao and Drasgow, 2019).

Generally speaking, to answer a FC personality inventory, individuals must choose the option that best or worst describes them among several options with a similar loading in social desirability or social preference. FC personality inventories assume that, due to the difficulty that the individuals have in choosing the option that is the most socially acceptable, they will tend to choose the alternative that best describes them, reducing, therefore, the effect of faking on the personality scores (Jackson et al., 2000; Christiansen et al., 2005; Converse et al., 2006). Therefore, FC inventories differ from SS inventories in that individuals have to make a choice between different alternatives, and they do not rate each alternative as occurs in SS measures (Salgado and Táuriz, 2014; Salgado et al., 2015; Salgado, 2017).

However, FC personality inventories are not a single category as three types of FC scores can be distinguished based on the metric properties of the measures: normative, ipsative, and quasi-ipsative (or partially ipsative) FC inventories (e.g., Clemans, 1966; Hicks, 1970; Meade, 2004; Salgado and Táuriz, 2014). In the case of normative FC scores, individuals must choose between options that represent examples of a single dimension. In other words, normative formats contain one-dimensional items. The normative FC scores allow comparisons at both intra-individual and between-individual levels. Ipsative FC scores are characterized by the fact that all the alternatives in each item must be rated. Consequently, there is a dependence between the various dimensions, in the sense that the level of the individual in one personality dimension is dependent on the level of the same individual in other assessed dimensions. Hence, this type of FC only allows to compare the scores at intra-individual level and it only shows the relative relevance of each factor for the individual (Clemans, 1966). Quasi-ipsative formats do not meet all the criteria of pure ipsative measures. According to Hicks (1970) and Meade (2004), quasi-ipsative scores are defined by the following characteristics: (a) the results for each personality factor vary between individuals over a certain range of scores; (b) the scores do not add up to the same constant for all individuals, even though these inventories have some properties in common with the ipsative measures; and (c) the increase in the score in one personality factor does not necessarily produces a decrease in the score in other factors. Therefore, quasi-ipsative FC scores allow both intra-individual and between-individual comparisons. In summary, quasi-ipsative formats share properties with normative and pure ipsative measures.

The importance of the distinctions between these categories or groups of FC personality inventories is particularly relevant in connection with their predictive validity. Over the years, FC inventories have been criticized mainly for their psychometric properties because of the degree of dependence between scores which could affect their validity for predicting organizational outcomes (e.g., Zavala, 1965; Hicks, 1970; Baron, 1996; Bartram, 1996; Christiansen et al., 1998; Jackson et al., 2000). However, recent empirical evidence has shown that FC inventories are valid procedures to predict performance in academic and organizational contexts, particularly in the case of the quasi-ipsative FC inventories. The meta-analyses of Bartram (2005, 2007), Fisher et al. (2019), Salgado and Táuriz (2014), and Salgado et al. (2015) showed that FC personality inventories are valid predictors of organizational and academic performance.

Some relevant contributions appeared in the last few years onto the theoretical foundations and advantages of using the classical test theory (CTT) vs. the item response theory (IRT) to develop FC personality inventories (Hontangas et al., 2015, 2016; Morillo et al., 2019).

Several FC personality inventories were developed in the last two decades based on the CTT, for example, the Employee Screening Questionnaire (ESQ, Jackson et al., 2000), the QI5F_tri (Salgado and Lado, 2018), the GPP (Gordon, 1993), the IPIP-MFC (Heggestad et al., 2006b). The CTT-based process of constructing FC personality inventories is similar to creating SS personality inventories. The critical difference between FC and SS personality inventories is that in the case of FC personality inventories all the statements included in an item are similarly rated in terms of social desirability and preference. Another important feature is that the FC personality inventories can be classified into two big categories (a) with algebraical dependence among the scales (e.g., ipsative FC personality inventories and some QI personality inventories) and (b) without algebraic dependence among the scales (e.g., normative FC and some QI personality inventories). Algebraical dependence means that the scores for a particular scale depend to some extent on the scores to other scales. This fact affects internal consistency coefficients because it vulnerates the principle of independence of the errors. In this case, test–retest reliability is the most adequate estimate of the reliability (Heggestad et al., 2006a). The scales developed without algebraical dependence do not suffer this limitation as the scores in a scale are totally independent of other scales. Therefore, internal consistency coefficients can be calculated as the errors are independent (Salgado and Lado, 2018).

Concerning IRT models, two predominant classes exist to characterize the process of responding to a single personality statement: (1) dominance models and (2) ideal-point models. These models reflect different assumptions about the response process underlying an examinee's decision to agree or disagree with an item. Dominance models assume an examinee will endorse a personality statement if his or her trait level is greater than the “location” of the item. Dominance models are predicated upon the seminal work of Rensis Likert. They have been used almost exclusively in psychology to create measures of constructs, including personality traits (Chernyshenko et al., 2007). A dominance model aims to establish the likelihood that an examinee would endorse an item as a function of the item characteristics, most notably the *location* of the item (i.e., the point on the trait continuum at which the item demonstrates optimal measurement) and the examinee's level of that trait. In technical terms, this is called an item response function (IRF). The IRF indicates that someone with a trait level higher than that optimally measured by the item is likely to respond affirmatively to the item. Someone with a trait level lower than the level optimally measured by the item is expected to disagree with the item. The critical point is that a dominance model predicts that every examinee with a trait level higher than the one measured by the item is expected to respond affirmatively.

Ideal-point models have been suggested to be more theoretically and empirically appropriate for personality measurement (see Drasgow et al., 2010 for a non-technical summary). Ideal-point models are based on the seminal work by Thurstone (e.g., 1927; 1928). At their core, ideal-point models postulate that an examinee endorses items based on the *distance* between his/her trait level and the location of the item, with a smaller distance reflecting a greater probability of endorsement. In the case of ideal-point models, the IRF shows that someone with a trait level much higher than that of the item is just as likely to disagree with the item as someone with a trait level much lower than that of the item. In contrast, examinees with trait levels very close to the item's level are likely to agree with the item. Just like with the dominance model, an examinee with a trait level lower than that represented by the personality item would disagree with the item. However, the important difference is that the ideal-point model also accounts for the fact that someone may differ from “above” because the personality item's trait level is not extreme enough to accurately represent him or her. The accuracy of ideal-point models has also received significant empirical support. Research has shown that ideal-point models provide an as good or better fit to personality data than dominance models (Stark et al., 2005, 2006). This was found to be the case even when examining personality assessments built on the assumptions of dominance models. This was likely because ideal-point models are flexible enough to accurately model *both* items exhibiting ideal-point characteristics and those exhibiting dominance characteristics. Additionally, ideal point models allow improved measurement precision across the entire range of personality levels by using items that more accurately assess moderate trait levels (Chernyshenko et al., 2007). These types of moderate items are often rejected during test development, relying on dominance models because they often display a poor fit.

In connection with the IRT models of FC personality inventories is also relevant to mention the work by Maydeu-Olivares and his colleagues (Maydeu-Olivares and Bockenholt, 2005; Maydeu-Olivares and Brown, 2010; Brown and Maydeu-Olivares, 2011, 2012). Based on the Thurstonian models, Maydeu-Olivares and his colleagues developed a method to recover normative scores from an ipsative FC personality inventory.

At present, the empirical evidence comparing the validity of CTT-based FC personality inventories vs. the validity of IRT-based FC personality inventories is scarce. For example, Brown and Maydeu-Olivares (2013) found that the validity of an IRT-based scoring system of FC personality inventory was slightly higher than the validity of the CTT-based scoring system (0.09 higher on average). Recently, Lee et al. (2018) investigated the criterion validity of two IRT-based methods and a CTT-based method of scoring a quasi-ipsative FC personality inventory. They found that CTT, the simplest method, was more effective than the IRT-based methods. Fisher et al. (2019) conducted a small-scale meta-analysis (*N* = 611, *K* = 11) using a single quasi-ipsative FC inventory. Fisher et al. (2019) found that the CTT-based scoring system showed a higher predictive validity than the IRT-based scoring system (0.38 vs. 0, respectively).

In summary, the most recent meta-analytic research on the validity of FC inventories reached three important conclusions. First, the quasi-ipsative format proved to be a more valid predictor of performance than the other FC formats and the SS personality inventories. Second, of all the personality factors, quasi-ipsative FC measures of conscientiousness are the best personality predictor of job and academic performance. The meta-analytic research also found that quasi-ipsative FC measures of personality were the best predictors of performance for all occupational categories.

### Forced-Choice Inventories and Faking

Regarding the evidence of the effectiveness of FC inventories in reducing the effects of faking, the results of primary studies have been inconsistent. Some studies found that both FC and SS personality inventories were affected by faking to a similar degree (e.g., Heggestad et al., 2006a). Other research has suggested that faking is considerably reduced when FC inventories are used. For example, Jackson et al. (2000) did not find significant differences in the correlations between the low and high motivation conditions for faking when a FC format was used. Christiansen et al. (2005) found that faking was lower for FC inventories (*d* = 0.26) compared to SS inventories (*d* = 0.96). Likewise, Bowen et al. (2002) found less faking in an ipsative FC inventory than in the SS version of the same inventory (*d* = 0.24 vs. *d* = 0.41). Nevertheless, the meta-analyses carried out on this issue support the robustness of FC inventories against faking.

Chronologically, Nguyen and McDaniel (2001), Adair (2014), and Cao and Drasgow (2019) provided meta-analytic evidence of the faking resistance of FC inventories. Nguyen and McDaniel (2001) found effects sizes for FC inventories which were smaller than those reported by Viswesvaran and Ones (1999) for SS measures, with average values of *d* = 0.40 and *d* = 0.58 for the between-subject designs and within-subject designs, respectively. Adair (2014) found effect sizes ranging from *d* = 0.15, for agreeableness, to *d* = 0.70, for conscientiousness. Therefore, this meta-analytic evidence showed that, although individuals motivated to fake can distort the scores in the FC inventories and they are not totally robust against faking, the effects of faking were smaller for FC inventories than for SS inventories.

Cao and Drasgow (2019) carried out the largest meta-analysis so far about effects of faking on FC personality inventories. The results of this meta-analysis suggested that the use of FC inventories considerably reduces the effects of faking. They found that conscientiousness (*d* = 0.23) followed by agreeableness (*d* = 0.19) and extraversion (*d* = 0.16) were the personality factors most affected by distortion, but the effect sizes obtained were smaller than those obtained for SS inventories (Viswesvaran and Ones, 1999; Birkeland et al., 2006).

Cao and Drasgow (2019) also examined whether the FC format (pick, mole, or rank), the question design (one-dimensional or multidimensional), and the study design (experimental contexts and applied contexts) affect the level of faking. They found that the pick format (*d* = 0.05) and multidimensional items (*d* = 0.03) were more effective against faking than the mole format (*d* = 0.37) and the one-dimensional items (*d* = 0.13). Regarding the type of design, they found that experimental studies (induced faking) showed larger effect sizes on average than field studies, which corresponds with Birkeland, Manson, Kisamore, Brannick and Smith (2006) findings on the effects of the study design. However, the effect sizes were significantly smaller for FC inventories than those found for SS inventories. In summary, the main conclusion of these three meta-analyzes is that FC inventories are a suitable procedure for reducing the pernicious effects of faking in personnel selection processes and hiring decisions.

### Limitations of Previous Meta-Analyses

Despite the relevant contributions of these meta-analyses, they have some methodological limitations that must be considered in order to give a full account of their contribution to the literature.

An initial limitation of the prior meta-analyses relates to the characteristics of the samples and the coding data. For instance, the number of independent samples in Nguyen and McDaniel's (2001) meta-analysis is very small. In addition, they did not distinguish between studies with faking good and faking bad manipulations, grouping data from both conditions in a single category. This could have led to erroneous conclusions in their work.

The compilation and codification of the primary studies included in Cao and Drasgow's (2019) meta-analysis also have some inconsistencies. First, Cao and Drasgow (2019) misclassify the types of FC inventories from the primary studies in several cases. For example, they classified as normative (pick) FC inventories that are quasi-ipsative (mole) FC formats (see Supplementary Material). Second, they duplicated the samples (the number of participants) in all within-subject designs. Within-subject designs are characterized by the fact that the same individuals respond in both honest and faking conditions, that is, the same sample answer the FC inventory twice, once under each condition. However, Cao and Drasgow (2019) interpret each condition as a different sample and add the individuals from both honest and faking conditions, thus doubling the real sample size of these studies. Third, the meta-analysis did not mention how they coded the data in relation to the various personality dimensions. They only report an average effect size of the personality scales instead of one for each personality factor. In the Supplementary Material we included the discrepancies we found in the coding data of Cao and Drasgow's (2019) meta-analysis.

The meta-analysis of Cao and Drasgow (2019) has another critical limitation. The accumulated sample of their meta-analysis came largely from a primary study of the TAPAS (Drasgow et al., 2012), which has an important methodological limitation. This study contributed over 120,000 individuals which fully determined their meta-analytic findings. However, the characteristics of the experimental context of Drasgow et al.'s (2010) study do not conform to the faking vs. honest research conditions. Trent et al. (2020) pointed out that Drasgow et al. (2012) administered the TAPAS under honest conditions, concurrently with other measures that were part of a real selection process. Nevertheless, the participants were not informed of the research-only purpose of the personality test administration. As a result, according to Trent et al. (2020), the participants likely believed that their responses were being used for selection purposes. Also, the participants might be motivated to respond in a socially desirable manner. Thus, Trent et al. (2020) sustain that the small effect sizes obtained in this study might not be related to the use of FC measures but rather to the fact that both groups would be similarly motivated to fake. As a consequence, the inclusion of the study of Drasgow et al. (2012) could severely condition Cao and Drasgow's (2019) meta-analytic findings.

A second limitation of the previous meta-analysis is related to the presence of artifacts (for instance, sampling error, measurement error, or range restriction) that could bias the estimates of the effect size, leading to erroneous conclusions (Hunter and Schmidt, 1987a,b; Schmidt and Hunter, 2015). Previous meta-analyses did not examine the effect of some of these artifacts. Specifically, Adair (2014), Cao and Drasgow (2019), and Nguyen and McDaniel (2001) carried out “bare-bones” meta-analyses. Therefore, they only considered the pervasive effect of the sampling error. Hence, these meta-analyses did not correct the observed effect sizes for measurement error and range restriction. Consequently, their results could be biased by the effect of these artifacts.

A third limitation is that no previous meta-analyses analyzed the joint effect of the potential moderating variables. For instance, Adair (2014) analyzed the moderating effect of the design type on the faking resistance of the FC inventories, but Adair (2014) only provided results for the experimental settings. Cao and Drasgow (2019) analyzed separately the moderating effect of the format of FC and the type of design. However, none of these meta-analyses have carried out a hierarchical analysis of the moderators that have been studied independently in the research about faking. Previous results have shown that both the experimental design and the FC format could independently affect the magnitude of the effect sizes, so it is necessary to examine their joint effect and as well their independent effect.

Taken together, these limitations suggest that a new meta-analysis to expand on the previous research and to overcome the limitations mentioned above could shed further light on the resistance of the FC personality inventories to faking.

### Aims of the Study and Research Hypotheses

This meta-analysis has two main objectives. First, to estimate the degree of resistance of FC inventories to the effects of faking. Second, to analyze the moderating effects of the study design (i.e., within-subject, between-subject, applicant, and incumbent), and the effects of the FC format (i.e., ipsative, quasi-ipsative, and normative).

Regarding the resistance of FC inventories to faking, available meta-analytic evidence suggests that these personality instruments are effective in reducing faking (Nguyen and McDaniel, 2001; Adair, 2014; Cao and Drasgow, 2019). As FC formats force the individual to choose between equally desirable items, this should lead to a reduction in the response distortion. Therefore, based on these previous findings the following hypothesis is proposed:

*Hypothesis 1: FC personality inventories are resistant to the effects of faking, as illustrated by a smaller Cohen's d than the one found for SS inventories in previous meta-analyses*.

The second issue is whether the format of FC is a moderating variable of the resistance of these inventories to faking. Previous research has shown that the metric differences between each of the formats could affect their validity (see, for instance, Salgado and Táuriz, 2014). As faking is a mechanism that reduces the validity of personality inventories, and because the quasi-ipsative FC format showed higher validity than the other two FC formats, we advance the next hypothesis:

*Hypothesis 2: The quasi-ipsative FC format is more resistant to the effects of faking than the ipsative and normative FC formats*.

As previous meta-analytic evidence showed that the study design can affect the magnitude of faking (e.g., Viswesvaran and Ones, 1999; Birkeland et al., 2006; Salgado, 2016), the effects of faking being greater for the within-subject designs, for the studies conducted in experimental settings (as opposed to real selection settings), and for applicants (vs. incumbents), we advance the next three hypotheses:

*Hypothesis 3a: Study design is a moderator of the effects of faking on personality measures*.
*Hypothesis 3b: The magnitude of faking is higher in experimental contexts than in real personnel selection contexts*.
*Hypothesis 3c: The magnitude of faking is higher for within-subject designs (vs. between-subject designs), in experimental contexts, and for samples of applicants (vs. incumbents) in applied contexts*.

## Method

### Literature Research

An extensive literature search was carried out. The aim was to identify the largest possible number of studies that directly analyzed the faking resistance of FC personality inventories or studies that published data that would allow us to study this effect (e.g., comparison of applicant with incumbent samples). We used five strategies to achieve this goal. First, we conducted a computer-based literature search (until February 2020) in the following databases and meta-databases: EBSCO Host, PsycInfo, ResearchGate, ScienceDirect, Taylor & Francis, Wiley Online Library, Google, and Google Scholar. We used combinations of the following keywords: forced-choice, forced-choice inventory, forced-choice format, ranking format, faking, impression management, response distortion, social desirability, score distortion, personality, Five-Factor Model and Big Five. Second, we carried out manual article-by-article searches in the following scientific journals (between January 1990 and February 2020): European Journal of Work and Organizational Psychology, Human Performance, International Journal of Selection and Assessment, Journal of Applied Psychology, Journal of Clinical Psychology, Journal of Educational Measurement, Journal of Personality and Social Psychology, Journal of Work and Organizational Psychology, Personality and Individual Differences, Personnel Psychology, and Psychological Reports). Third, we reviewed the reference section of the articles found in the previous strategies. Fourth, we examined the reference sections of previous meta-analyses (Nguyen and McDaniel, 2001; Adair, 2014; Salgado and Táuriz, 2014; Cao and Drasgow, 2019) to identify articles not located in the previous approaches. Finally, we obtained papers and additional information from five researchers who were contacted by us. The studies included in their meta-analysis are disclosed in the Supplementary Material.

### Inclusion Criteria

Each study was carefully examined to determine whether it was suitable to be included in the meta-analysis. We used the following four criteria. First, the studies had to report the effect size value or provide data to allow its calculation. Second, the studies whose response-instructions did not conform to the faking or honest conditions were excluded from the meta-analysis. Specifically, we did not include (a) research that used other strategies concurrently with faking instructions (for example, specific warning instructions about the process) and (b) studies whose experimental context cast doubt on the sincerity of the participants when responding under honest instructions. Third, only studies that used FC personality inventories in one of their three formats (ipsative, quasi-ipsative or normative) were included in the meta-analysis. Finally, we only included primary studies whose personality measures were based on the Five-Factor Model (FFM) or that could be classified using this model. In this second case, we used the following classification strategy: two researchers experts in the area of personality at work served as coders that independently classified each scale into one of the personality factors, based on the definitions of the Big Five given by Costa and McCrae (1992) and Salgado (1998), among other sources. Moreover, the coding list used by Hough and Ones (2001); Salgado (2003); Birkeland et al. (2006), and Salgado and Táuriz (2014) were also checked. If the coders agreed on a dimension, the scale was coded in that dimension. The disagreements were solved by a discussion until the researchers agreed on a dimension. All the scales were assigned to a single dimension. Table 1 presents the list of the personality inventories included in the meta-analysis.

**Table 1:** Forced-choice personality inventories included in the meta-analysis.

ADEPT-15—Adaptive Employee Personality Test
ATHURE—Test Human Relations
Dunnette Adjective Checklist
EPPS—Edwards Personal Preference Schedule
Forced Choice Goldberg's Factors Markers
ESQ_FC—Employee Screening Questionnaire
FC-FFM—Forced Choice Five Factor Markers
GPI—Gordon Personal Inventory
GPP—Gordon Personal Profile
GSDI—Ghiselli Self-Description Inventory
IPIP-FCM—IPIP Multidimensional Forced Choice
MBTI—Myers-Briggs Type Indicator
NCAPS—Navy Computer Adaptive Personality Scales
OPQ—Occupational Personality Questionnaire
PAPI—Perception and Preference Inventory
Pensacola Z
QI5F-tri—Quasi-ipsative Forced Choice Questionnaire
Q-Sort
SFCAS—Short Forced Choice Anxiety Scale
SIV—Survey Interpersonal Values
TAPAS—Tailored Adaptive Personality Assessment System
TDOT—Thorndike Dimensions of Temperament
WPQ—Work Preference Questionnaire

**Figure 1:**
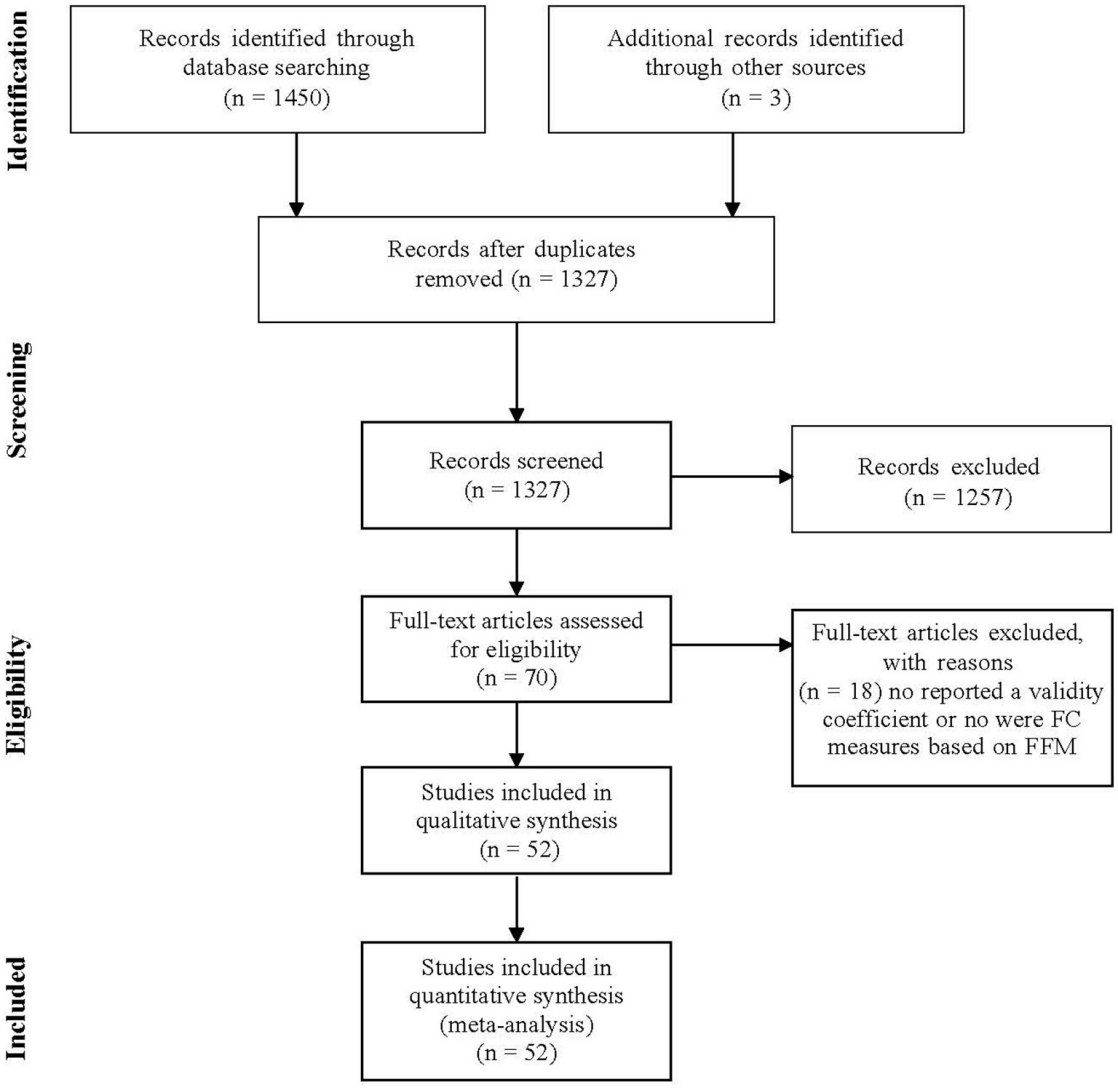
PRISMA flow diagram of excluded and included articles.

The search produced 52 documents with 82 independent samples. The cumulated sample size was 106,266 subjects. According to the Preferred Reporting Items for Systematic Reviews and Meta-analysis (PRISMA) guidelines (Moher et al., 2009), the PRISMA How diagram is shown in Figure 1. Additionally, in the Supplementary Material, we report the studies included in the meta-analyses.

### Coding Procedure

For each study, we recorded the following information: (a) study characteristics (i.e., author, year, title, publication type; publication name); (b) sample characteristics, i.e., size (initial and final size), type (students, applicants, motivated-to-fake incumbents, and nonmotivated-to-fake incumbents); (c) study context (i.e., experimental or real selection context); (d) design characteristics (i.e., within-subject design or between-subject design); (e) personality variables, i.e., measure type (ipsative, quasi-ipsative, normative), personality factors, reliability; and (f) effect size (i.e., Cohen's *d* or data that allowed its calculation).

The two authors participate actively in the literature search process and in the data coding. To obtain inter-coder agreement estimates, two authors coded independently the following variables: (a) study year; (b) publication type (published vs. unpublished); (c) sample size; (d) sample type; (e) effect size; (f) FC-questionnaire type (i.e., ipsative, quasi-ipsative, normative); (g) design type (i.e., between-subject, within-subject); (h) reliability of the Big Five personality dimension; and (i) study context. To establish the level of inter-coder agreement, we identified the total number of data points and the number of disagreements. The overall level of agreement was 95.6%. Twenty-three disagreements were resolved by referring back to the studies and discussion between the two researchers until consensus was reached.

### Decision Rules

We applied several decision rules due to the peculiarities of some of the studies included in this research. These rules concern three aspects: (1) duplicate samples; (2) results obtained from the comparisons with a normative sample; and (3) effect size of measures not based on the FFM.

*1. Duplicate samples*. The main consequence of including duplicate samples is the overestimation of the effect size in the study, which, in turn, causes alterations in other relevant statistics of the meta-analysis such as sampling error variance and the observed standard deviation of the effect size (Schmidt, 2008). For this reason, a typical practice is to analyze the presence of non-independent samples and to apply an adjustment method in the data. In our case, we applied two procedures depending on the study characteristics: (a) when the studies were duplicated, that is, the sample and experimental conditions were the same, only the most recently published study was included in the database; (b) when the studies reported estimators of the effect size in different faking conditions with the same sample, the conditions of each case were analyzed for inclusion in the meta-analysis. If the studies reported effects sizes under both general and specific faking instructions (for example, faking that affects a single characteristic or faking for a specific job profile), only the effect size of the general condition was included in the meta-analysis. In the case in which there were considered to be no differences between the experimental designs, the average effect size was calculated for inclusion in the meta-analysis.

In the hierarchical meta-analyses, the average effect sizes were coded separately to examine the effect of possible moderators and to obtain a more accurate estimate of the relationship. As these meta-analyses are specific, there is no duplication of the samples.

*2. Normative samples*. When the primary studies reported data from the faking condition only, data from the normative sample of the personality inventory (usually manual data) were used as the honest condition to calculate the effect size. Due to the fact that the magnitude of the normative sample was typically very large, only the sample which participated in the experimental study was included as the value of n. This strategy was also applied to those primary studies that reported estimators of the effect size as a result of the comparison with normative samples.

*3. Personality measures (compounds)*. Several studies assessed personality with non-FFM based inventories, but those scales could be categorized into the Big Five personality dimensions. In those cases, we followed two strategies. First, when the study reported correlations between the personality scales or these correlations could be obtained from normative data, the compound correlation was calculated using the formula given by Schmidt and Hunter (2015). Schmidt and Hunter (2015) point out that the application of the compound formula allows for a more accurate estimation of the effect size than if an average correlation between the variables is calculated. To apply these formulas the effect sizes must be reported as Pearson coefficients (*r*). As many of the primary studies reported the correlations as a Cohen's *d*, a double transformation was done to calculate the compounds: first, each Cohen's *d* was transformed into *r*, to calculate the correlation of the compound (*r*), and then, this value was transformed into a Cohen's *d* again. The formulas reported by Hunter and Schmidt (2004) were used to perform the effect size transformations. Second, when the primary studies did not report the correlations between the different variables (scales) of each personality compound, the average effect size was calculated.

### Analysis of Moderators

Moderators are variables that may affect the correlation between two other variables (Schmidt and Hunter, 2015). Therefore, when meta-analyses are carried out, it is important to consider the possible presence of moderator variables that may affect the results. Schmidt and Hunter (2015) suggest that to study the effect of a moderator the most appropriate method is to divide the meta-analyses into subgroups.

In the present study, we considered two moderator variables. The first moderator was the study design. In accordance with faking research, we distinguished four study types: within-subject studies, between-subject studies, applicant studies, and incumbent studies. We considered it relevant to examine these four study types separately because the degree of motivation to fake may be different in each type. As we stated in hypotheses 3a, 3b, and 3c incumbents were less motivated to fake than applicants, and experimental studies showed higher faking than studies conducted in real workplace contexts.

The second moderator was the type of FC inventory. Based on the scores they produced, we considered three FC types: ipsative, quasi-ipsative, and normative. Therefore, we analyzed the three FC types separately to determine if the metric characteristics of each of them affect the results differently.

Finally, we considered the joint effect of both moderators on faking estimates. According to Schmidt and Hunter (2015), when the researcher cannot assume that the moderator variables are independent a hierarchical meta-analysis should be conducted to discover the true influences and interaction of the moderator variables. A hierarchical meta-analysis results in a more precise analysis of the relationships between the variables because it allows us to determine the joint effect of the moderators. In this meta-analysis, we were able to find primary studies to estimate the degree of faking for the combination of FC type and study type for the following eight categories: (1) ipsative-within-subject design; (2) ipsative-between-subject design; (3) ipsative-applicant design; (4) ipsative-incumbent design; (5) quasi-ipsative-within-subject design; (6) quasi-ipsative-between-subject design; (7) quasi-ipsative-applicant design; and (4) quasi-ipsative-incumbent design. We did not find enough studies to conduct a hierarchical meta-analysis for the normative FC personality inventories.

### Effect Size Estimation

In this study, a meta-analysis of differences between conditions has been carried out. Hence, if the primary studies reported an effect size expressed in Cohen's *d*, the estimate was directly included in the meta-analysis. When the primary studies did not report an effect size but data that allowed its estimation or statistics that could be transformed into a Cohen coefficient (*d*), we proceeded to its calculation. In the first case, the data of mean, SD and sample size reported by the studies allowed for the calculation of an experimental research effect size (i.e., Cohen's *d*) through the formula given by Schmidt and Hunter (2015), p. 277). In the second case, we transformed statistics *t*, into a Cohen's *d* using the formulas proposed by Rosenthal (1994), p. 239).

Finally, the directionality of the relationships was checked and reversed when necessary to maintain the direction of the results constant, such that the negative sign meant that a lower score had been obtained in the faking condition than in the honest condition.

### Reliability

In order to perform measurement error corrections, we developed an empirical distribution of the reliability for each Big Five personality dimension. We used the reliability coefficients provided in the primary studies supplemented by coefficients published in the manuals (when the study did not report reliability data) to construct those reliability distributions. Descriptive statistics of the distributions appear in Table 2. All the values included in the distributions are internal consistency coefficients expressed as Cronbach's alpha. None of the meta-analyses carried out so far made corrections for measurement error. Therefore, the estimates reported in Table 2 on the distributions of the reliabilities of the analyzed variables are a unique contribution of this study.

**Table 2:** Reliability distributions for the variables (Big Five).

	***K***	** $\bar{\text{r}}$ *_***xx***_***	***SD_***rxx***_***	**Range**
**Within-subject designs**				
Emotional stability	9	0.60	0.25	0.32/0.87
Extraversion	13	0.76	0.10	0.65/0.89
Openness to experience	12	0.76	0.10	0.57/0.87
Agreeableness	12	0.70	0.12	0.49/0.83
Conscientiousness	17	0.72	0.10	0.49/0.87
**Between-subject designs**				
Emotional stability	15	0.78	0.13	0.47/0.92
Extraversion	16	0.80	0.07	0.69/0.90
Openness to experience	15	0.78	0.11	0.57/0.98
Agreeableness	14	0.71	0.15	0.27/0.83
Conscientiousness	27	0.71	0.11	0.49/0.87
**Applicant designs**				
Emotional stability	4	0.73	0.18	0.47/0.87
Extraversion	6	0.79	0.08	0.69/0.85
Openness to experience	3	0.74	0.21	0.57/0.98
Agreeableness	3	0.52	0.26	0.27/0.78
Conscientiousness	8	0.68	0.13	0.49/0.84
**Incumbent designs**				
Emotional stability	3	0.82	0.06	0.71/0.82
Extraversion	4	0.82	0.06	0.73/0.85
Openness to experience	2	0.81	0.01	0.80/0.81
Agreeableness	2	0.75	0.12	0.66/0.83
Conscientiousness	7	0.79	0.10	0.64/0.87
**Ipsative inventories**				
Emotional stability	1	0.77	–	0.77
Extraversion	4	0.74	0.11	0.66/0.89
Openness to experience	4	0.71	0.07	0.66/0.77
Agreeableness	4	0.64	0.12	0.49/0.77
Conscientiousness	4	0.74	0.10	0.66/0.86
**Quasi-ipsative inventories**				
Emotional stability	23	0.71	0.20	0.32/0.92
Extraversion	25	0.79	0.08	0.65/0.90
Openness to experience	23	0.78	0.11	0.57/0.98
Agreeableness	22	0.71	0.14	0.27/0.83
Conscientiousness	39	0.71	0.11	0.49/0.87

*K = number of reliability coefficients; $\bar{r}$_xx_ = average value of reliability distribution; SD_rxx_ = standard deviation of distribution reliability values; Range = minimum and maximum values of distribution reliability*.

### Meta-Analytic Methods and Software

To conduct this meta-analysis, we applied the methods of meta-analysis of differences developed by Schmidt and Hunter (2015; see also Hunter and Schmidt, 1990, 2004), using the software created by Schmidt and Le (2014), V2.0) that applies this methodology and permits to control the impact of artifacts on the effect size.

## Results

The results of the meta-analyses carried out are described below. Table 3 reports the results for the Big Five across the three types of FC personality inventories and Table 4 the meta-analytic results for the Big Five across the four types of study designs. Tables 5–**7** report the hierarchical meta-analyses for the Big Five across the combination of three FC inventory formats and the four study types. From left to right, each of the tables contains the number of independent samples (*K*), the accumulated sample size (*N*), the sample size weighted mean effect size (*d*_*w*_), the observed variance ($S_{obs}^{2}$), the observed standard deviation (*SD*_*obs*_), and the variance explained for sampling error (*S*^2^_e_). The next three columns report the true effect size (δ), the standard deviation of δ (*SD*_δ_), and the percentage of variance accounted for by the artifactual errors (% *VE*). The last two columns report the 90% credibility value based on δ (*VC*_δ_ 90%) and the 95% confidence interval of δ (*IC*_δ_ 95%).

**Table 3:** Meta-analysis of the faking resistance of forced-choice inventories by type of forced-choice format.

	***K***	***N***	***d_***w***_***	*** ${\text{S}}_{obs}^{2}$ ***	***SD_***obs***_***	*** ${\text{S}}_{e}^{2}$ ***	**δ**	***SD*_**δ**_**	***%VE***	**90% CV_**δ**_**	**95% CI*_**δ**_***
**Ipsative FC Inventories**											
Emotional stability	13	1,958	−0.14	0.1313	0.3624	0.0268	−0.16	0.3684	20	0.31	−0.38/0.07
Extraversion	17	2,090	0.27	0.1391	0.3730	0.0331	0.32	0.3774	24	−0.17	0.11/0.52
Openness to experience	17	2,090	0.05	0.1926	0.4389	0.0328	0.06	0.4730	17	−0.55	−0.19/0.31
Agreeableness	17	2,090	−0.23	0.2168	0.4657	0.0330	−0.28	0.5350	15	0.40	−0.56/−0.01
Conscientiousness	17	2,090	0.27	0.2244	0.4738	0.0331	0.32	0.5098	15	−0.34	0.05/0.58
**Quasi-ipsative FC Inventories**											
Emotional stability	37	8,604	0.29	0.1331	0.3648	0.0175	0.35	0.4051	15	−0.17	0.20/0.49
Extraversion	36	14,527	−0.01	0.0561	0.2368	0.0100	−0.01	0.2422	18	0.30	−0.10/0.08
Openness to experience	35	99,772	−0.18	0.0159	0.1260	0.0014	−0.21	0.1360	10	−0.04	−0.26/−0.16
Agreeableness	34	94,827	0.06	0.0170	0.1305	0.0014	0.07	0.1487	9	−0.12	0.01/0.12
Conscientiousness	56	102,671	0.36	0.0237	0.1540	0.0022	0.43	0.1708	13	0.21	0.38/0.48
**Normative FC Inventories**											
Emotional stability	4	1,169	−0.17	0.1339	0.3659	0.0138	−0.17	0.3465	10	0.27	−0.53/0.19

*The negative sign in the scores means a lower score in the faking condition. K = number of independent samples; N = accumulated sample size; d_w_ = sample size weighted mean effects size expressed in Cohen's d; ${\text{S}}_{obs}^{2}$ = observed variance; SD_obs_ = observed standard deviation; ${\text{S}}_{e}^{2}$ = sampling error variance; δ = true effect size expressed in Cohen's d; SD_δ_ = standard deviation of δ; %VE = percentage of variance accounted for artifactual errors; 90% CV_δ_ = 90% credibility value based on δ; 95% CI_δ_ = 95% confidence interval of δ*.

**Table 4:** Meta-analysis of the faking resistance of the forced-choice inventories by type of design.

	***K***	***N***	***d_***w***_***	*** ${\text{S}}_{obs}^{2}$ ***	***SD_***obs***_***	*** ${\text{S}}_{e}^{2}$ ***	**δ**	***SD*_**δ**_**	***%VE***	**90% CV_**δ**_**	**95% CI*_**δ**_***
**Within-subject designs**											
Emotional stability	26	4,237	0.32	0.1855	0.4307	0.0251	0.42	0.5204	17	−0.25	0.20/0.63
Extraversion	27	4,288	0.14	0.1441	0.3796	0.0255	0.16	0.3961	18	−0.35	−0.01/0.32
Openness to experience	27	3,984	0.16	0.1305	0.3613	0.0275	0.18	0.3694	21	−0.29	0.02/0.34
Agreeableness	27	3,984	0.02	0.2218	0.4710	0.0274	0.02	0.5303	12	−0.66	−0.19/0.24
Conscientiousness	37	4,622	0.40	0.2458	0.4957	0.0330	0.47	0.5457	14	−0.23	0.28/0.66
**Between-subject designs**											
Emotional stability	28	6,153	0.09	0.1435	0.3789	0.0150	0.10	0.4081	11	−0.42	−0.05/0.26
Extraversion	26	10,946	−0.01	0.0458	0.2139	0.0085	−0.01	0.2159	19	0.26	−0.11/0.08
Openness to experience	25	96,537	−0.19	0.0113	0.1063	0.0010	−0.22	0.1145	11	−0.07	−0.27/−0.17
Agreeableness	25	91,804	0.05	0.0145	0.1202	0.0011	0.06	0.1383	7	−0.12	0.00/0.12
Conscientiousness	37	100,179	0.36	0.0178	0.1334	0.0015	0.42	0.1482	13	0.24	0.37/0.48
**Applicant samples**											
Emotional stability	12	2,510	−0.10	0.0842	0.2902	0.0192	−0.12	0.3010	23	0.26	−0.32/0.07
Extraversion	14	8,090	−0.06	0.0289	0.1697	0.0070	−0.07	0.1668	24	0.15	−0.17/0.04
Openness to experience	11	93,807	−0.21	0.0025	0.0504	0.0005	−0.24	0.0452	40	−0.18	−0.28/−0.21
Agreeableness	11	88,851	0.05	0.0026	0.0509	0.0005	0.07	0.0631	23	−0.01	0.03/0.11
Conscientiousness	16	95,104	0.35	0.0032	0.0564	0.0007	0.43	0.0465	54	0.37	0.39/0.46
**Incumbent samples**											
Emotional stability	7	986	−0.22	0.0930	0.3050	0.0288	−0.25	0.2864	31	0.11	−0.51/0.00
Extraversion	7	509	0.21	0.0423	0.2058	0.0559	0.23	0.0000	100	0.23	0.09/0.40
Openness to experience	5	460	0.30	0.0930	0.3049	0.0443	0.34	0.2460	48	0.02	0.04/0.64
Agreeableness	6	479	−0.25	0.1803	0.4246	0.0510	−0.29	0.4169	28	0.25	−0.68/0.11
Conscientiousness	7	509	−0.22	0.0663	0.2576	0.0560	−0.25	0.1142	85	−0.10	−0.46/−0.03

*The negative sign in the scores means a lower score in the faking condition. K = number of independent samples; N = accumulated sample size; d_w_ = sample size weighted mean effects size expressed in Cohen's d; ${\text{S}}_{obs}^{2}$ = observed variance; SD_obs_ = observed standard deviation; ${\text{S}}_{e}^{2}$ = sampling error variance; δ = true effect size expressed in Cohen's d; SD_δ_ = standard deviation of δ; %VE = percentage of variance accounted for artifactual errors; 90% CV_δ_ = 90% credibility value based on δ; 95% CI_δ_ = 95% confidence interval of δ*.

**Table 5:** Hierarchical meta-analysis of the faking resistance of ipsative forced-choice inventories.

	***K***	***N***	***d_***w***_***	*** ${\text{S}}_{obs}^{2}$ ***	***SD_***obs***_***	*** ${\text{S}}_{e}^{2}$ ***	**δ**	***SD*_**δ**_**	***%VE***	**90% CV_**δ**_**	**95% CI*_**δ**_***
**IPSATIVE FC**											
**Emotional stability (ES)**											
ES—Within-subject designs	2	234	0.06	0.0745	0.2729	0.0347	0.06	0.1995	47	−0.20	−0.32/0.44
ES—Between-subject designs	4	588	0.21	0.0274	0.1656	0.0276	0.24	0.0000	100	0.24	0.06/0.43
ES—Applicants	5	912	−0.37	0.0551	0.2348	0.0224	−0.37	0.1809	41	−0.13	−0.57/−0.16
ES—Incumbents	2	224	−0.35	0.1551	0.3939	0.0365	−0.35	0.3445	24	0.09	−0.90/0.19
**Extraversion (EX)**											
EX—Within-subject designs	6	366	0.87	0.1231	0.3508	0.0729	1.02	0.2512	62	0.69	0.69/1.34
EX—Between-subject designs	4	588	0.13	0.1028	0.3207	0.0275	0.15	0.3129	27	−0.25	−0.21/0.51
EX—Applicant	5	912	0.14	0.0264	0.1639	0.0221	0.14	0.0691	82	0.05	−0.00/0.28
EX—Incumbent	2	224	0.20	0.0067	0.0819	0.0361	0.20	0.0000	100	0.20	0.08/0.31
**Openness to experience (OE)**											
OE—Within-subject designs	6	366	0.38	0.6656	0.8156	0.0678	0.45	0.9266	10	−0.73	−0.33/1.23
OE—Between-subject designs	4	588	0.01	0.1167	0.3417	0.0274	0.01	0.3406	23	−0.43	−0.38/0.39
OE—Applicant	5	912	−0.09	0.0272	0.1649	0.0221	−0.09	0.0717	81	−0.00	−0.24/0.05
OE—Incumbent	2	224	0.21	0.0035	0.0594	0.0361	0.21	0.0000	100	0.21	0.12/0.29
**Agreeableness (A)**											
A—Within-subject designs	6	366	−0.49	0.7264	0.8523	0.0686	−0.63	1.0457	10	−0.71	−1.51/0.25
A—Between-subject designs	4	588	−0.27	0.2363	0.4891	0.0276	−0.31	0.5206	12	0.36	−0.85/0.24
A—Applicant	5	912	−0.09	0.0054	0.0737	0.0221	−0.09	0.0000	100	−0.09	−0.15/−0.02
A—Incumbent	2	224	−0.26	0.0000	0.0008	0.0362	−0.26	0.0000	100	−0.26	−0.26/−0.26
**Conscientiousness (C)**											
C—Within-subject designs	6	366	1.08	0.0718	0.2680	0.0764	1.27	0.0000	100	1.27	1.02/1.53
C—Between-subject designs	4	588	0.18	0.0396	0.1989	0.0275	0.20	0.1252	70	0.04	−0.02/0.43
C—Applicant	5	912	0.15	0.0883	0.2972	0.0221	0.15	0.2573	25	−0.18	−0.11/0.41
C—Incumbent	2	224	−0.32	0.0003	0.0170	0.0364	−0.32	0.0000	100	−0.32	0.34/−0.30
**QUASI-IPSATIVE FC**											
**Emotional stability (ES)**											
ES—Within-subject designs	16	3,042	0.45	0.1288	0.3589	0.0217	0.61	0.4280	25	0.07	0.37/0.86
ES—Between-subject designs	10	3,709	0.30	0.1055	0.3249	0.0109	0.34	0.3501	11	−0.10	0.11/0.58
ES—Applicant	7	1,598	0.05	0.0394	0.1985	0.0176	0.05	0.1743	45	−0.17	−0.12/0.23
ES—Incumbent	4	255	−0.27	0.1927	0.4390	0.0641	−0.31	0.4053	33	0.21	−0.79/0.18
**Extraversion (EX)**											
EX—Within-subject designs	14	2,998	0.01	0.0928	0.3047	0.0188	0.02	0.3148	20	−0.39	−0.17/0.20
EX—Between-subject designs	9	4,378	0.07	0.0591	0.2431	0.0082	0.09	0.2520	14	−0.24	−0.09/0.26
EX—Applicant	8	6,866	−0.09	0.0244	0.1563	0.0047	−0.10	0.1584	19	0.11	−0.22/0.03
EX—Incumbent	5	285	0.22	0.0701	0.2648	0.0715	0.24	0.0000	100	0.24	−0.02/0.50
**Openness to experience (OE)**											
OE—Within-subject designs	16	2,903	0.14	0.0747	0.2733	0.0224	0.16	0.2553	30	−0.17	0.01/0.31
OE—Between-subject designs	10	3,738	0.11	0.1000	0.3162	0.0108	0.12	0.3440	11	−0.32	−0.10/0.35
OE—Applicant	6	92,895	−0.21	0.0022	0.0466	0.0003	−0.24	0.0427	38	−0.19	−0.29/−0.20
OE—Incumbent	3	236	0.39	0.1622	0.4027	0.0522	0.43	0.3696	32	−0.04	−0.07/0.94
**Agreeableness (A)**											
A—Within-subject designs	16	2,903	0.13	0.1041	0.3227	0.0224	0.15	0.3297	22	−0.27	−0.03/0.33
A—Between-subject designs	10	4,061	0.16	0.2594	0.5093	0.0099	0.19	0.5829	4	−0.56	−0.18/0.56
A—Applicant	5	87,627	0.05	0.0011	0.0331	0.0002	0.08	0.0459	27	0.02	0.03/0.13
A—Incumbent	3	236	−0.36	0.2506	0.5006	0.0520	−0.41	0.5166	21	0.25	−1.07/0.24
**Conscientiousness (C)**											
C—Within-subject designs	24	3,332	0.41	0.2288	0.4783	0.0298	0.49	0.5242	13	−0.19	0.26/0.71
C—Between-subject designs	18	5,214	0.53	0.2340	0.4837	0.0144	0.64	0.5593	17	−0.07	0.37/0.91
C—Applicant	9	93,840	0.35	0.0018	0.0429	0.0004	0.43	0.0230	81	0.40	0.39/0.46
C—Incumbent	5	285	−0.14	0.1045	0.3233	0.0713	−0.16	0.2055	68	0.10	−0.48/0.16

*The negative sign in the scores means a lower score in the faking condition. K = number of independent samples; N = accumulated sample size; d_w_ = sample size weighted mean effects size expressed in Cohen's d; ${\text{S}}_{obs}^{2}$ = observed variance; SD_obs_ = observed standard deviation; ${\text{S}}_{e}^{2}$ = sampling error variance; δ = true effect size expressed in Cohen's d; SD_δ_ = standard deviation of δ; %VE = percentage of variance accounted for artifactual errors; 90% CV_δ_ = 90% credibility value based on δ; 95% CI_δ_ = 95% confidence interval of δ*.

Psychometric meta-analyses are typically carried out to examine the extent to which validity generalization exists. In other words, to estimate the degree to which the average effect size obtained in a meta-analysis can be expected to be found in other studies not included in that meta-analysis (Schmidt and Hunter, 1977). However, in this research, the aim is to study the validity generalization of faking, which is negative behavior. Therefore, the perspective used to interpret the results must be the opposite of the usual one, that is, the less generalization of faking behaviors involved the greater the resistance of the FC inventories to faking (the main hypothesis of this study). Thus, according to the hypothesis, it is expected that the intervals include zero, which would support the resistance of these personality instruments to faking. Credibility intervals estimate the true variability of the individual effect sizes across the distribution of studies. Thus, for example, a credibility value of 90% that includes zero indicates that 90% of the effect sizes cannot be considered statistically different from that value. Meanwhile, the confidence interval provides an estimate of the variability of the average effect size. Thus, a 95% confidence interval that includes zero indicates, with a confidence of 95%, that the average effect size is not different from zero (Judge and Bono, 2001; Schmidt and Hunter, 2015).

### Meta-Analytic Results for the Big Five Across the FC Personality Types

As can be seen in Table 3, the results considered as a whole show a clear pattern. First, the *d* estimates are relatively small or even negative for the three types of FC personality inventories, the observed *d* ranging from −0.23 to 0.36. This finding indicates that the FC personality inventories are robust against faking. Secondly, the *d* estimates are noticeably different across the Big Five personality dimensions. This finding shows that faking affects the Big Five differently in workplace situations. For instance, conscientiousness is more affected by faking than openness and agreeableness. Thirdly, the *d* estimates are not consistent across the three types of FC inventories. This finding might be partially due to the different nature of the studies grouped in each FC inventory type.

Examining the results according to the Big Five personality dimensions, emotional stability is the only personality dimension that has been assessed with the three types of FC inventories. The results show that the faking effects were fully controlled in the cases of ipsative and normative FC inventories (−0.14 and −0.17, respectively) and small in the case of quasi-ipsative FC inventories (0.29). The results for extraversion show that the faking effects were also small or inexistent (−0.27 and −0.01 for ipsative and quasi-ipsative FC inventories, respectively). The effects of faking on openness to experience are also irrelevant (0.05 and −0.18 for ipsative and quasi-ipsative FC inventories, respectively). This was also the case for the faking effects on agreeableness (−0.23 and 0.06, for ipsative and quasi-ipsative FC inventories, respectively). Finally, conscientiousness is the only personality dimension modestly affected by faking (0.27 and 0.26 for ipsative and quasi-ipsative FC inventories, respectively).

### Meta-Analytic Results for the Big Five Across the Design Types

The results for the effects of faking on the combination of the Big Five and the four types of designs are reported in Table 4. The findings of this meta-analysis also show a clear pattern.

First, the *d* estimates are very small or even negative for all the Big Five-study type combinations, except for the conscientiousness-applicant combination on which faking showed a modest effect (*d* = 0.35). Again, this finding indicates that FC personality inventories are generally robust against faking.

Secondly, the *d* estimates are noticeably different across the four study-design types, but they show a noticeable consistency among the designs. In 18 out of 20 values the pattern is that within-sample designs showed greater *d* than between-sample designs, between-sample designs show greater *d* than applicant studies, and applicant studies greater *d* than incumbent studies. The only two exceptions are for extraversion and openness in the case of incumbent studies. Considered as a whole, these comparisons reveal that the extent of faking is greater for experimental studies (i.e., within-sample and between-sample studies than for real workplace studies (i.e., applicant and incumbent).

Also, the findings of this meta-analysis reveal that faking has no important practical effect on emotional stability, extraversion, openness, and agreeableness, as showed by the magnitude of the *d* values for the applicant samples. Only conscientiousness is modestly affected by faking in applied contexts.

The examination of the findings at the level of the Big Five personality dimensions shows that the values ranged from 0.32 for the within-sample studies to −0.22 for the incumbent studies. Similar results were found for extraversion, with *d*-values ranging from 0.21 to −0.06 (for incumbent studies and applicant studies, respectively). In relation to openness, the *d*-values ranged from 0.30 to −0.21 (again for incumbent studies and applicant studies, respectively). For agreeableness, the *d*-values ranged from 0.05 to −0.25 (for between-sample studies and incumbent studies, respectively). Finally, the range of *d*-values for conscientiousness was from 0.40 to −0.22, for the within-sample studies and incumbent studies, respectively.

From an applied perspective and, particularly, from the point of view of the potential effects of faking on personnel selection processes, the most relevant findings are the ones obtained for the studies with applicants. As can be seen in Table 4, faking has no practical effects for emotional stability, extraversion, openness to experience, and agreeableness (*d*-values ranging from −0.21 to 0.05, for openness and agreeableness, respectively). Faking has only a small-to-modest effect for conscientiousness (*d* = 0.35).

In summary, the overall results of this first meta-analysis give support to Hypotheses 1 and Hypotheses 3a and Hypotheses 3b of this study.

### Results of the Hierarchical Meta-Analyses for the Big Five Personality Dimensions Across the Combination of FC Type-Study Design

Table 5 shows the results of the effects of faking for the Big Five when the two moderators are combined. Schmidt and Hunter (2015) pointed out that the examination of the effects of the moderator combination in hierarchical meta-analysis must be conducted to identify if they interact. We have been able to conduct the hierarchical meta-analysis for the combination of ipsative and quasi-ipsative FC inventories and the study design but not for the normative FC inventories as we did not find primary studies for this last combination.

The combination of the two moderators shows that, in general, the faking effects on emotional stability, extraversion, openness, and agreeableness are small or irrelevant in the case of real workplace settings (i.e., samples of incumbents and applicants). For conscientiousness, the results show a small-to-modest effect. The effect of faking is particularly noticeable for the combinations of FC inventories and within-subject studies.

Next, we examined the results at the Big Five level. The findings for emotional stability showed a moderate effect size for quasi-ipsative FC formats in both the experimental designs (0.47 and 0.30 for within-subject and between-subject designs, respectively), However, faking has no practical effects for applicants and incumbents in both ipsative and quasi-ipsative FC formats.

Regarding extraversion, with the exception of Cohen's *d* for the ipsative format in within-subject designs (*d* = 0.87), the results did not show relevant differences between the ipsative and quasi-ipsative formats, the *d*-values being small in all cases.

In the case of openness to experience, the largest effect sizes were for ipsative formats in within-subject designs and for studies with real-incumbent samples that used quasi-ipsative inventories (0.38 and 0.39, respectively)

Agreeableness showed negative effect sizes in six out eight cases, and the effect size was very small in the additional two cases (0.17 and 0.05, respectively). Therefore, faking was not shown to have a relevant effect for agreeableness.

The results for conscientiousness showed some differences between the ipsative and quasi-ipsative formats of the FC inventories. For instance, the effect size was larger for the combinations of ipsative FC-within-subject studies and ipsative-incumbent studies than for the quasi-ipsative combinations (1.08 and 0.32 vs. 0.41 and−0.14, while the effect size was larger for the combination of quasi-ipsative FC-between-subject and quasi-ipsative-applicant than for the ipsative combinations (0.53 and 0.35 vs. 0.18 and 0.18, respectively).

In summary, these hierarchical meta-analyses carried out to analyze the joint effect of the type of design and the FC format showed that both variables interact as moderators of the effects of faking on FC inventories. Consequently, Hypothesis 2 and Hypothesis 3 (a and b) were confirmed. However, as can be seen in Table 5, the percentage of explained variance was small and the values of SD were again relevant in most cases, which suggests that other moderator variables may be affecting the results.

### Faking Resistance Comparison of FC and SS Personality Inventories

Tables 6, 7 present the overall results of three prominent investigations that have analyzed the susceptibility of SS personality inventories to faking behavior (Viswesvaran and Ones, 1999; Birkeland et al., 2006; Salgado, 2016) and the overall meta-analytical results obtained in the current study for FC formats. Table 6 summarizes the results obtained in experimental contexts, while Table 7 shows the findings in real-life contexts of personnel selection.

**Table 6:** Comparison between the meta-analyses of the resistance to faking of FC and SS personality inventories in experimental contexts.

	**VandO-SS**			**Salgado-SS**			**Average-SS**			**FC-IP**			**FC-QIP**		
	***K***	***N***	***d_***w***_***	***K***	***N***	***d_***w***_***	***K***	***N***	***d_***w***_***	***K***	***N***	***d_***w***_***	***K***	***N***	***d_***w***_***
**Within-subject designs**															
Emotional stability	29	921	0.93	4	427	0.24	33	1,348	0.59	2	234	0.06	16	3,042	0.45
Extraversion	10	391	0.54	5	607	0.21	15	998	0.38	6	366	0.87	14	2,998	0.01
Openness to experience	9	259	0.73	5	607	0.02	14	866	0.38	6	366	0.38	16	2,903	0.14
Agreeableness	14	408	0.47	5	607	0.55	19	1,015	0.51	6	366	−0.49	16	2,903	0.13
Conscientiousness	24	723	0.89	5	607	0.13	29	1,330	0.51	6	366	1.08	24	3,332	0.41
**Between-subject designs**															
Emotional stability	17	1,357	0.64							4	588	0.21	10	3,709	0.30
Extraversion	15	1,122	0.63							4	588	0.13	9	4,378	0.07
Openness to experience	11	614	0.65							4	588	0.01	10	3,738	0.11
Agreeableness	17	1,009	0.48							4	588	−0.27	10	4,061	0.16
Conscientiousness	19	2,650	0.60							4	588	0.18	18	5,214	0.53

*K = number of independent samples; N = accumulated sample size; dw = sample size weighted mean effects size expressed in Cohen's d; V&O-SS = Viswesvaran and Ones (1999); Salgado-SS = Salgado (2016); FC-IP = Forced-choice ipsative inventories; FC-QIP = forced-choice quasi-ipsative inventories*.

**Table 7:** Comparison between the meta-analyses of the resistance of faking of FC and SS personality inventories in applied contexts.

	**Salgado-SS**			**Birkeland et al.-SS**			**Average-SS**			**FC-IP**			**FC-QIP**		
	***K***	***N***	***d_***w***_***	***K***	***N***	***d_***w***_***	***K***	***N***	***d_***w***_***	***K***	***N***	***d_***w***_***	***K***	***N***	***d_***w***_***
**Applicant designs**															
Emotional stability	11	32,599	0.23	25	35,210	0.44	36	67,809	0.34	5	912	−0.37	7	1,598	0.05
Extraversion	11	32,599	0.17	29	71,841	0.11	40	104,440	0.14	5	912	0.14	8	6,899	−0.09
Openness toexperience	12	32,917	0.49	20	60,261	0.13	32	93,178	0.31	5	912	−0.09	6	92,895	−0.21
Agreeableness	10	31,203	0.17	20	43,968	0.16	30	47,171	0.17	5	912	−0.09	5	87,627	0.05
Conscientiousness	13	33,002	0.29	27	88,266	0.45	40	121,268	0.37	5	912	0.15	9	93,840	0.35
**Incumbent designs**															
Emotional stability	18	5,467	0.34							2	224	−0.35	4	255	−0.27
Extraversion	20	5,798	0.49							2	224	0.20	5	285	0.22
Openness to experience	20	5,645	0.38							2	224	0.21	3	236	0.39
Agreeableness	18	5,467	0.30							2	224	−0.26	3	236	−0.36
Conscientiousness	21	5,896	0.27							2	224	−0.32	5	285	−0.14

*K = number of independent samples; N = accumulated sample size; d_*w*_ = sample size weighted mean effects size expressed in Cohen's d; Salgado-SS = Salgado (2016); Birkeland et al.-SS = Birkeland et al. (2006); FC-IP = Forced-choice ipsative inventories; FC-QIP = forced-choice quasi-ipsative inventories*.

With regard to the results obtained in experimental contexts (Table 6), if we compare the effect sizes of the SS inventories and FC inventories for the within-subject designs, the second type showed smaller magnitudes than the first type, with only two exceptions for ipsative FC inventories, the effect sizes of extraversion (0.87 vs. 0.38) and conscientiousness (1.08 vs. 0.51). Regarding the between-subjects designs, the results indicated substantially smaller effect sizes for both FC formats than for SS inventories, but, in particular, the quasi-ipsative format showed in both designs to be more robust against the effects of faking than the SS inventories.

Results concerning applied contexts (Table 7) showed that the magnitude of faking is smaller in this type of design than in experimental contexts. The effect sizes found were small or moderate in all cases (<0.49) and showed smaller differences between the effect sizes of both types of personality instruments SS and FC inventories. However, as can be seen, FC inventories proved to be, on average, more resistant to faking behavior. Hence, these findings suggest that FC personality inventories are a more useful personality instrument for controlling the effects of faking than SS inventories.

## Discussion

This study reports on a comprehensive meta-analysis on the resistance to the effects of faking of FC inventories. Overall, FC inventories showed resistance to faking. In fact, comparing the effect sizes obtained in this study with those found by Viswesvaran and Ones (1999) for SS personality measures, it can be seen that, in general, the effect sizes provided by the current meta-analysis are smaller than those obtained by Viswesvaran and Ones (1999), indicating that FC inventories show more resistance to faking than SS measures. Therefore, these results give support to Hypothesis 1. Although, this was not the only contribution of this research.

In relation to hierarchical meta-analyzes, the results obtained allow us to affirm that the study design and the type of FC inventory (ipsative, quasi-ipsative or normative) affect the magnitude of faking. In the case of the study design, this meta-analysis showed substantial differences in effect sizes between experimental designs and applied contexts. Experimental designs showed higher magnitudes of faking, regardless of the type of measure used. Therefore, the results obtained suggest that the type of study design affects the results. Consequently, these results support previous findings found by Salgado (2016) for SS measures when comparing experimental studies and correlational (real sample) studies. Thus, Hypothesis 3 and Hypothesis 3a also get empirical support. This is the second contribution of this meta-analysis.

Moreover, the findings showed that the within-subject designs for ipsative measures obtained the largest effect sizes in the experimental designs, while they were significantly smaller even irrelevant for applicant samples and incumbent samples in the applied contexts, the smallest effect sizes being those found for incumbent designs in which faking is only observed in two factors, extraversion and openness to experience, and, in both cases, it was of little magnitude. However, it should be noted that the typical “before-after” design used in the faking study is not powerful enough to rule out the effects of transient measurement errors, suggesting that a part of the effect size attributed to faking in such designs could be a consequence of transient error. These results suggest that the effects of faking are practically irrelevant in applied contexts (e.g., personnel selection) when FC measures of personality are used. In this sense, Hypothesis 3b would also be confirmed. This is also a unique contribution of the current study.

Consequently, it can be posited that the magnitude of the faking may be, in part, a laboratory phenomenon. On the one hand, the largest effect sizes found in laboratory studies (experimental contexts) do not occur in real-life personnel selection. On the other hand, the experimental designs used did not control for the effect of the transient measurement error, whose effects are assigned to faking. This is the fourth unique contribution of our study.

Regarding the type of FC inventory, our meta-analytic results revealed that both ipsative and quasi-ipsative formats reduce the effects of faking. However, if we focus on the magnitudes of Cohen's *d*, it is the quasi-ipsative FC format that has shown the greatest resistance to this phenomenon obtaining, on average, smaller effects size than the ipsative FC inventories. Therefore, Hypothesis 2 is also supported. This is another important contribution of this study because it shows the robust resistance of this inventory to the effects of faking.

Finally, two important variables, which the scientific literature has identified as moderators of faking behaviors, were analyzed: the type of study design and the type of FC inventory. To this purpose, a hierarchical meta-analysis of these moderators has been conducted, the first to date on this topic. This is, therefore, another unique contribution of this meta-analysis.

### Implications for the Theory, Practice and Future Research

The findings have implications for the theory of faking at work. The psychometric theory of faking effects suggests that factor structure, construct validity, criterion validity, and incremental validity of personality inventories are negatively affected by faking (Salgado, 2016; Otero et al., 2020; Martínez et al., 2021a,b). In connection with this, the findings of this meta-analytic study imply that other characteristics being equal, FC personality inventories would be more robust against the reduction of the validity levels than their counterpart SS personality inventories would be. In the second place, as several researchers have posited (McFarland and Ryan, 2006; Tett et al., 2012; Tett and Simonet, 2021), the opportunity to fake is one of the main determinants of actual faking. About this last point, the findings showed that the characteristics of FC personality inventories clearly reduce the opportunity to fake by increasing the difficulty to answer in a socially desirable way, which subsequently diminishes applicant's motivation to fake. A third implication of the findings is that given that faking is positively related to deviant behavior on the job (Tett and Simonet, 2021), FC personality inventories by reducing faking, contribute to decrease potential counterproductive behaviors at work. A final implication is that as inventories susceptible to faking are more likely to yield adverse impact (Christiansen et al., 2021; Tett and Simonet, 2021), the FC personality inventories, by controlling faking, reduce adverse impact.

Form the applied point of view, the meta-analytic results showed that the effects of faking are not substantial when FC measures of personality are used. Hence, it suggests that FC format reduces the faking effects on personality measures in personnel selection contexts. This fact has important implications for applied contexts.

Firstly, these results showed that FC inventories are useful procedures for controlling faking. For this reason, the use of these personality measures in personnel selection processes instead of SS personality measures is strongly recommended.

Second, regarding the FC format, although it can be concluded that all types of FC measures are resistant to faking, the quasi-ipsative format has shown itself to be the most resistant to faking, especially if we focus on the conscientiousness factor. In conclusion, the use of quasi-ipsative FC personality measures are recommended in applied contexts (e.g., personnel selection). Thus, they are a suitable alternative to SS personality measures for the evaluation of personality.

Regarding future research, it is recommended that more primary studies be carried out in order to obtain more robust results for the meta-analyses with a small number of studies, such as in our case the hierarchical meta-analyses for the normative format.

Likewise, new primary studies should be carried out to investigate other moderator variables of faking that could not be analyzed in this meta-analysis, such as the type of occupation, the literature has shown that individuals can modify their responses depending on the job on which are based (Sackett et al., 1989); or the personal characteristics of the participants, several theories of the antecedents of faking have proposed that the abilities of each individual can also affect their intention to distort (Paulhus, 1991; McFarland and Ryan, 2000, 2006). In addition, there may be other variables that we did not consider in this research but that it could be necessary to investigate in order to obtain more robust results regarding the effects of faking.

### Limitations of the Study

Like other meta-analytic studies, this research has some limitations. The first limitation of this study is that we were not able to conduct a hierarchical meta-analysis of normative FC inventories, because this category contains only a small number of studies. For this reason, new research should expand this meta-analysis in order to compare the resistance of all formats of FC inventories and, consequently, to reach more precise conclusions about the resistance of FC inventories to faking. The second limitation of this study is that it was not possible to analyze other variables that could be moderators of the effects of faking. Thus, for example, the type of employment about which the subjects are thinking when they answer the personality inventories (in experimental contexts or in personnel selection processes) is a variable that must also be analyzed to know if it affects the results.

## Conclusions

Faking is one of the main problems in the evaluation of personality, especially in the contexts of personnel selection. Individuals that fake can affect the whole evaluation process, modifying the applicants ranking and causing erroneous hiring decisions. FC inventories are a method of personality assessment that could reduce the effects of faking.

This study presents a comprehensive meta-analysis on the faking resistance of FC inventories. The results have provided several unique contributions: (1) FC inventories show resistance to faking behavior; (2) the magnitude of faking is higher in experimental contexts than in real-life selection processes, suggesting that the effects of faking may be, in part, a laboratory phenomenon; and (3) quasi-ipsative FC inventories are more resistant to faking than the other FC formats. These findings, therefore, show the robustness of quasi-ipsative FC inventories for controlling against the effects of faking and, consequently, have theoretical and practical relevance for the assessment of personality.

## Data Availability Statement

The original contributions presented in the study are included in the article/Supplementary Material, further inquiries can be directed to the corresponding author/s.

## Author Contributions

All authors listed have made a substantial, direct and intellectual contribution to the work, and approved it for publication.

## Funding

The research reported in this article was partially supported by Grant PID2020-116409GB-100 from the Spanish Ministry of Science and Innovation.

## Conflict of Interest

The authors declare that the research was conducted in the absence of any commercial or financial relationships that could be construed as a potential conflict of interest.

## Publisher's Note

All claims expressed in this article are solely those of the authors and do not necessarily represent those of their affiliated organizations, or those of the publisher, the editors and the reviewers. Any product that may be evaluated in this article, or claim that may be made by its manufacturer, is not guaranteed or endorsed by the publisher.
