# Supplementary material for: A Meta-Analysis of the Faking Resistance of Forced-Choice Personality Inventories
Source: Front Psychol. 2021 Sep 29;12:732241. doi: 10.3389/fpsyg.2021.732241 (PMC8511514; doi:10.3389/fpsyg.2021.732241)
Supplement: Supplementary file 1 [file Data_Sheet_1.docx]

Supplementary Material

*Table 1. Values of the Primary Studies Compared to Coding Data of the Meta-analysis of Cao and Drasgow (2019)*

| Reference | Design  Primary studies | *N*  primary studies | *N*  Cao & Drasgow (2019) | Format FC  primary study | Format FC  Cao & Drasgow (2019) |
| --- | --- | --- | --- | --- | --- |
| Anderson et al. (1984) | B | 36 | 36 | No FC | N/A |
| Boyce, Conway & Caputo (2015) | W | 88 | 176 | Q | N |
|  | W | 108 | 216 | Q | N |
|  | W | 106 | 212 | Q | N |
|  | B | 86974 | 86974 | Q | N |
|  | B | 5268 | 5269 | Q | N |
| Braun (1962a) | W | 19 | 38 | Q | Q |
|  | W | 53 | - | Q | - |
|  | W | 11 | - | Q | - |
|  | W | 39 | - | Q | - |
| Braun (1963a) | W | 26 | 52 | Q | I |
| Braun (1963b) | W | 24 | 48 | Q | Q |
|  | W | 25 | 50 | Q | Q |
| Braun (1965a) | W | 69 | 138 | Q | Q |
|  | W | 44 | 88 | Q | Q |
| Braun & Farrell (1974) | B | 61 | 61 | Q | Q |
|  | B | 90 | 90 | Q | Q |
| Braun & Lafaro (1967) | W | 86 | 172 | Q | Q |
| Braun & Lafaro (1969) | W | 11 | 22 | No FC | N/A |
|  | W | 20 | 40 | No FC | N/A |
|  | W | 19 | 38 | No FC | N/A |
| Christiansen et al. (2005) | B | 350 | 350 | Q | I |
|  | B | 122 | - | Q | - |
| Converse et al. (2010) | B | 107 | 107 | Q | I |
| Converse et al. (2010) | B | 113 | 113 | Q | I |
|  | B | 100 | 100 | Q | I |
|  | B | 104 | 104 | Q | I |
| Dicken (1959) | W | 19 | 38 | I | I |
| Drasgow et al. (2012) | B | 117620 | 117620 | Q | N |
| Dunnette et al. (1962) | W | 62 | 124 | Q | I |
|  | W | 63 | 126 | Q | I |
|  | W | 96 | 166 | Q | I |
|  | W | 64 | 113 | Q | I |
| Fluckinger | B | 435 | 435 | Q | I |
| Gordon & Stapleton (1956) | W | 88 | 209 | Q | I |
|  | W | 121 | 242 | Q | I |
| Griffith, Peterson, Quist, Benda & Evans (2008) | W | 21250 | 42500 | N | N/A |
| Guan (2015) | W | 1130 | 2260 | Q | N |
| Hedberg (1962) | W | 59 | 118 | Q | I |
| Heggestad et al. (2006) | B | 590 | 575 | Q | I |
| Hirsh & Peterson (2008) | B | 203 | 203 | I | N/A |
| Jackson et al. (2000) | W | 106 | 212 | Q | I |
| Kaess & Witryol (1957) | B | 507 | 507 | N | I |
|  | B | 264 | 264 | N | I |
|  | B | 361 | 361 | N | I |
| Kanning & Kuhne | B | - | 110 | FC | N/A |
| Kirchner (1962) | B | 166 | 166 | I | I |
|  | B | 115 | 115 | I | I |
| Kirchner et al. (1960) | B | 362 | 1122 | I | I |
| Larson et al. (2013) | B | 253 | 253 | Q | Q |
| Longstagg & Jurgensen | W | 41 | 82 | FC | I |
|  | W | 37 | 74 | - | I |
|  | W | 68 | 136 | - | I |
| Mahar et al. (2006) | W | 25 | 50 | I | N/A |
|  | W | 24 | 24 | I | N/A |
|  | W | 24 | 24 | I | N/A |
|  | W | 24 | - | I | - |
|  | W | 24 | - | I | - |
| Mahar et al. (1995) | W | 44 | 88 | I | N/A |
|  | W | 44 | - | I | - |
|  | W | 108 | 216 | Q | N |
|  | W | 106 | 212 | Q | N |
|  | B | 86974 | 86974 | Q | N |
|  | B | 5268 | 5269 | Q | N |
| Mudd |  | 120 | 120 | SS | N/A |
| Norman (1963) | W | 456 | 456 | Q | I |
|  |  | - | 456 | - | I |
| Rusmore (1956) | W | 81 | 162 | Q | Q |
| Schwab (1971) | W | 11 | 22 | Q | Q |
| Schwab (1971) | B | 22 | - | Q | - |
|  | W | 20 | 40 | Q | Q |
|  | B | 40 | - | Q | - |
| Shipley, Gray & Newbert | N/A | 784 | 784 | No FC | N/A |
|  | N/A | - | 815 | - | N/A |
| Underhill et al. (2008) | W | 75 | 148 | I | N/A |
| Vasilopoulos et al. (2006) | B | 167 | 167 | Q | Q |

*Note*: W = within-subjects design; B = between-subjects design; *N* = sample size; I = ipsative FC inventory; Q = quasi-ipsative FC inventory; N = normative FC inventory; SS = single-stimulus inventory.

*Table 2. Codes and Input Values for the Primary Studies Included in the Meta-analyses*

| Reference | *N* | FC Format | Design | Type  Sample | ES | α_es_ | EX | α_ex_ | OE | α_o_ | A | α_a_ | C | α_c_ | |
| --- | --- | --- | --- | --- | --- | --- | --- | --- | --- | --- | --- | --- | --- | --- | --- |
| Alonso (2011) | 215 | I | W | S | 0.14 | *-* | 0.82 | *-* | 0.63 | *-* | -1.11 | *-* | 0.90 | *-* | |
| Anderson, Sison, & Wester (1984) | 36 | N | B | S | - | *-* | - | *-* | - | *-* | - | *-* | - | *-* | |
| Antler, Zaretsky, & Ritter (1967) | 276 | Q | B | S | - | *-* | 0.35 | *.85* | - | *-* | - | *-* | -0.31 | *.87* | |
| Bass (1957) | 736 | Q | B | A | 0.17 | *.82* | 0.10 | *.85* | - | *-* | - | *-* | 0.17 | *.84* | |
| Bowen, Martin, & Hunt (2002) | 294 | I | B | S | 0.13 | *.77* | 0.45 | *.77* | 0.34 | *.77* | -0.02 | *.77* | 0.36 | *.77* | |
| Braun (1962a) | 19 | Q | W | S | - | *-* | - | *-* | 0.85 | *.87* | 0.31 | *.83* | 2.07 | *.65* | |
| Braun (1962b) | 61 | Q | W | S | 1.10 | *-* | - | *-* | - | *-* | - | *-* | 0.71 | *-* | |
|  | 53 | Q | W | S | 1.79 | *-* | - | *-* | - | *-* | - | *-* | 1.65 | *-* | |
|  | 11 | Q | W | S | 1.72 | *-* | - | *-* | - | *-* | - | *-* | 1.44 | *-* | |
|  | 39 | Q | W | S | 1.73 | *-* | - | *-* | - | *-* | - | *-* | 1.17 | *-* | |
| Braun (1963a) | 26 | Q | W | S | - | *-* | 0.61 | *-* | -1.51 | *-* | -0.62 | *-* | 1.36 | *-* | |
| Braun (1963b) | 24 | Q | W | S | - | *-* | - | *-* | 1.12 | *.87* | -0.02 | *.83* | 1.79 | *.65* | |
|  | 25 | Q | W | S | - | *-* | - | *-* | 1.33 | *.87* | 0.43 | *.83* | 1.30 | *.65* | |
| Braun (1963c) | 69 | Q | W | S | 1.01 | *.87* | 0.66 | *.85* | - | *-* | - | *-* | 1.14 | *.87* | |
|  | 44 | Q | W | S | 1.15 | *.87* | 0.44 | *.85* | - | *-* | - | *-* | 2.09 | *.87* | |
| Braun (1965) | 39 | I | W | S | - | *-* | 1.42 | *-* | -0.64 | *-* | -0.37 | *-* | 1.47 | *-* | |
| Braun & Alexander (1961) | 37 | N | W | S | 1.82 | *-* | - | *-* | - | *-* | - | *-* | - | *-* | |
| Braun & Farrell (1974) | 61 | Q | B | S | - | *-* | - | *-* | 0.81 | *.87* | 1.07 | *.83* | 1.21 | *.65* | |
|  | 90 | Q | B | S | 0.95 | *.87* | 0.82 | *.85* | - | *-* | - | *-* | 1.45 | *.87* | |
| Braun & Lafaro (1967) | 86 | Q | W | S | 0.47 | *-* | 0.59 | *-* | 0.25 | *-* | 1.12 | *-* | 1.03 | *-* | |
| Christiansen et al. (2005) | 350 | Q | B | S | - | *-* | 0.47 | *.84* | - | *-* | - | *-* | 0.40 | *.81* | |
|  | 122 | Q | B | S | - | *-* | - | *-* | - | *-* | - | *-* | 0.24 | *.76* |  |
| Converse et al. (2010) | 107 | Q | B | S | 0.94 | *.88* | - | *-* | - | *-* | - | *-* | 1.28 | *.66* |  |
|  | 113 | Q | B | S | 0.71 | *.88* | - | *-* | - | *-* | - | *-* | 1.05 | *.52* |  |
|  | 100 | Q | B | S | 0.86 | *.92* | - | *-* | - | *-* | - | *-* | 0.46 | *.69* |  |
|  | 104 | Q | B | S | 0.75 | *.88* | - | *-* | - | *-* | - | *-* | 0.51 | *.71* |  |
| Davis (2006) | 48 | Q | B | I | 0.06 | *.82* | 0.40 | *.85* | 0.95 | *.81* | 0.09 | *.83* | 0.35 | *.64* |  |
| Dicken (1959) | 19 | I | W | S | -0.90 | *-* | -0.35 | *-* | -0.99 | *-* | 0.91 | *-* | 1.54 | *-* |  |
| Dunnette et al. (1962) | 62 | Q | W | I | -0.73 | *-* | 0.58 | *-* | 0.73 | *-* | -1.20 | *-* | -0.27 | *-* |  |
|  | 63 | Q | W | A | -0.33 | *-* | 0.31 | *-* | 0.10 | *-* | -0.49 | *-* | -0.03 | *-* |  |
|  | 96 | Q | W | A | -0.40 | *-* | 0.64 | *-* | -0.05 | *-* | -0.20 | *-* | -0.42 | *-* |  |
|  | 64 | Q | W | A | -0.47 | *-* | 0.60 | *-* | 0.49 | *-* | -0.99 | *-* | -0.40 | *-* |  |
| Fineman (1975) | 40 | N | B | A | - | *-* | - | *-* | - | *-* | - | *-* | 0.08 | *.55* |  |
| Fluckinger (2010) | 435 | Q | B | S | -0.04 | *.82* | -0.04 | *.90* | -0.01 | *.70* | -0.06 | *.68* | 0.01 | *.77* |  |
| Ford & Gunderson (1970) | 232 | I | B | A | -0.37 | *-* | -0.04 | *-* | 0.17 | *-* | -0.04 | *-* | -0.14 | *-* |  |
|  | 37 | I | B | A | -0.15 | *-* | 0.06 | *-* | 0.00 | *-* | 0.17 | *-* | -0.04 | *-* |  |
| Furnham & Craig (1987) | 36 | I | B | S | 0.36 | *-* | -0.29 | *-* | -0.55 | *-* | -0.60 | *-* | 0.25 | *-* |  |
| Gordon & Stapleton (1956) | 88 | Q | W | A | 0.03 | *.87* | 0.16 | *.85* | - | *-* | - | *-* | 0.27 | *.84* |  |
|  | 121 | Q | W | A | 0.28 | *.74* | 0.00 | *.85* | - | *-* | - | *-* | 0.50 | *.68* |  |
| Guan (2015) | 1130 | Q | I | G | 0.39 | *-* | -0.22 | *-* | 0.11 | *-* | 0.31 | *-* | 0.06 | *-* |  |
| Hedberg (1962) | 59 | Q | W | S | - | *-* | 0.00 | *-* | -0.19 | *-* | 0.07 | *-* | 0.11 | *-* |  |
| Heggestad et al. (2006) | 590 | Q | B | S | 0.61 | *.80* | 0.33 | *.78* | 0.13 | *.78* | 0.07 | *.77* | 1.20 | *.82* |  |
| Helton & Street (1992) | 158 | I | B | I | -0.61 | *-* | 0.25 | *-* | 0.25 | *-* | -0.26 | *-* | -0.31 | *-* |  |
| Hirsh & Peterson (2008) | 203 | I | B | S | 0.40 | *-* | -0.21 | *-* | -0.26 | *-* | -0.84 | *-* | 0.01 | *-* |  |
| Jackson et al. (2000) | 106 | Q | W | S | - | *-* | - | *-* | - | *-* | - | *-* | 0.35 | *.69* |  |
| Kaess & Witryol (1957) | 507 | N | B | I | -0.14 | *-* | - | *-* | - | *-* | - | *-* | - | *-* |  |
|  | 264 | N | B | S | -0.23 | *-* | - | *-* | - | *-* | - | *-* | - | *-* |  |
|  | 361 | N | B | S | -0.37 | *-* | - | *-* | - | *-* | - | *-* | - | *-* |  |
| Kirchner (1962) | 166 | I | B | A | -0.07 | *-* | 0.16 | *-* | -0.16 | *-* | -0.09 | *-* | -0.11 | *-* |  |
|  | 115 | I | B | A | -0.06 | *-* | -0.07 | *-* | -0.10 | *-* | -0.05 | *-* | 0.02 | *-* |  |
| Kirchner et al. (1960) | 362 | I | B | A | -0.62 | *-* | 0.32 | *-* | -0.24 | *-* | -0.16 | *-* | 0.51 | *-* |  |
| Krug (1958) | 46 | Q | W | S | 1.04 | *-* | - | *-* | - | *-* | - | *-* | 1.33 | *-* |  |
| Larson et al. (2013) | 253 | Q | B | S | - | *-* | 0.36 | *0.87* | -0.39 | *0.67* | 1.27 | *0.73* | 1.40 | *.72* |  |
| Mahar et al. (2006) | 25 | I | W | S | - | *-* | 1.09 | *0.89* | 2.15 | *0.77* | 1.22 | *0.49* | 1.75 | *.86* |  |
|  | 24 | I | W | S | - | *-* | 0.16 | *0.66* | 1.82 | *0.66* | 1.94 | *0.66* | 2.59 | *.66* |  |
|  | 24 | I | W | S | - | *-* | 0.98 | *0.66* | 0.63 | *0.66* | 0.17 | *0.66* | 0.64 | *.66* |  |
|  | 24 | I | W | S | - | *-* | 0.34 | *0.66* | 2.36 | *0.66* | 2.77 | *0.66* | 1.92 | *.66* |  |
|  | 24 | I | W | S | - | *-* | 1.27 | *0.66* | 0.47 | *0.66* | 0.16 | *0.66* | 0.41 | *.66* |  |
| Mahar et al. (1995) | 44 | I | W | S | - | *-* | 1.55 | *0.66* | 0.00 | *0.66* | -0.04 | *0.66* | 0.23 | *.66* |  |
|  | 44 | I | W | S | - | *-* | 0.86 | *0.66* | -1.72 | *0.66* | -0.47 | *0.66* | 1.87 | *.66* |  |
| Martin & Theys (2019) | 88 | Q | W | G | 0.40 | *.32* | 0.31 | *.65* | 0.03 | *.72* | 0.09 | *.68* | 0.25 | *.72* |  |
|  | 108 | Q | W | G | 0.78 | *.32* | 0.47 | *.65* | 0.01 | *.72* | 0.46 | *.68* | 0.53 | *.72* |  |
|  | 106 | Q | W | G | 0.64 | *.32* | 0.55 | *.65* | 0.08 | *.72* | 0.48 | *.68* | 0.48 | *.72* |  |
|  | 86974 | Q | B | I | - | *-* | - | *-* | -0.20 | *.98* | 0.05 | *.27* | 0.35 | *.61* |  |
|  | 5268 | Q | B | I | - | *-* | -0.16 | *-* | -0.37 | *.68* | - | *-* | 0.42 | *.61* |  |
| Martínez (2019) | 653 | Q | B | S | 0.47 | *0.66* | -0.24 | *0.74* | 0.33 | *0.84* | -0.55 | *0.7* | 0.62 | *.75* |  |
|  | 490 | Q | W | S | 0.32 | *0.63* | -0.20 | *0.75* | 0.12 | *0.82* | -0.35 | *0.67* | 0.69 | *.78* |  |
|  | 176 | Q | B | S | 0.15 | *.54* | -0.12 | *.76* | 0.15 | *.74* | 0.06 | *.71* | 0.18 | *.80* |  |
| Mourer (1959) | 19 | Q | B | I | -1.43 | *0.82* | -0.35 | *0.85* | - | *-* | - | *-* | -1.12 | *.84* |  |
| Norman (1963) | 456 | Q | W | S | 0.00 | *-* | 0.02 | *-* | 0.06 | *-* | 0.01 | *-* | 0.00 | *-* |  |
| O´neil et al. (2016) | 185 | Q | B | S | - | *-* | 0.03 | *0.78* | - | *-* | 1.13 | *0.78* | 1.19 | *.78* |  |
|  | 176 | Q | B | S | - | *-* | 0.55 | *0.78* | - | *-* | 1.11 | *0.78* | 0.98 | *.78* |  |
|  | 312 | Q | B | S | - | *-* | -0.03 | *0.78* | - | *-* | 0.65 | *0.78* | 0.52 | *.78* |  |
| Pavlov et al. (2018) | 180 | Q | W | S | 0.69 | *-* | 0.60 | *-* | 0.63 | *-* | -0.14 | *-* | 0.68 | *-* |  |
| Rusmore (1956) | 81 | Q | W | S | - | *0.7* | 0.10 | *0.85* | - | *-* | - | *-* | - | *-* |  |
| Salgado & Lado (2018) | 126 | Q | B | I | -0.09 | *0.71* | 0.28 | *0.73* | 0.01 | *0.8* | -0.09 | *0.66* | -0.09 | *.80* |  |
| Schwab (1971) | 11 | Q | W | S | - | *-* | - | *-* | 0.62 | *-* | 0.28 | *-* | 0.60 | *-* |  |
|  | 22 | Q | B | S | - | *-* | - | *-* | 0.65 | *0.87* | 0.27 | *0.83* | 0.62 | *.65* |  |
|  | 20 | Q | W | S | - | *-* | - | *-* | 1.34 | *0.87* | 0.79 | *0.83* | 1.12 | *.65* |  |
|  | 40 | Q | B | S | - | *-* | - |  | 2.49 | *0.87* | 0.79 | *0.83* | 0.57 | *.65* |  |
| Sheppard et al. (1974) | 66 | I | B | I | 0.26 | *-* | 0.07 | *-* | 0.12 | *-* | -0.15 | *-* | -0.35 | *-* |  |
| Spector (1957) | 212 | N | B | S | - | *-* | - | *-* | - | *-* | 0.03 | *-* | - | *-* |  |
| Stollak (1965) | 56 | I | B | S | - | *-* | -0.01 | *-* | -0.45 | *-* | 0.71 | *-* | -0.20 | *-* |  |
| Trent et al. (2020) | 1341 | Q | B | S | 0.01 | *.47* | -0.04 | *.70* | -0.01 | *.57* | 0.07 | *.51* | 0.03 | *.49* |  |
|  | 430 | Q | W | A | 0.00 | *.47* | 0.12 | *.70* | 0.00 | *.57* | 0.15 | *.51* | 0.14 | *.49* |  |
| Underhill et al. (2008) | 75 | Q | W | S | - | *-* | 0.00 | *-* | -0.01 | *-* | 0.00 | *-* | 0.00 | *-* |  |
| Vasilopoulos et al. (2006) | 167 | Q | B | S | - | *-* | - | - | 0.19 | *0.66* | - | *-* | 0.27 | *.51* |  |

*Note*: *N* = sample size; I = ipsative FC inventory; Q = quasi-ipsative FC inventory; N = normative FC inventory; w= within-subjects design; B= between-subjects design; S= students samples; A= real job applicants samples; I = incumbents samples; G= general population; ES = emotional stability; α_ee_ = emotional stability reliability coefficient; EX = extraversion; α_ex_ = extraversion reliability coefficient; OE = openness to experience; α_ae_ = openness to experience reliability coefficient; A = agreeableness; α_a_ = agreeableness reliability coefficient; C = conscientiousness; α_c_ = conscientiousness reliability coefficient.

# References

Alonso, P. (2011). *Efectos de la deseabilidad social en tests normativos e ipsativos*. [Master’s thesis]. Spain: University of Santiago de Compostela.

Anderson, H. N., Sison, G., and Wester, S. (1984). Intelligence and dissimulation on the personal orientation inventory. *J. Clin. Psychol.*, 40, 1394–1398. doi:10.1002/1097-4679(198411)40:6<1394::AID-JCLP2270400620>3.0.CO;2-R

Antler, L., Zaretsky, H. H., and Ritter, W. (1967). The practical validity of the Gordon Personal Profile among United States and foreign medical residents. *J. Soc. Psychol.*, 72, 257–263. doi:10.1080/00224545.1967.9922323

Bass, B. M. (1957). Faking by sales applicants of a forced choice personality inventory*. J. Appl. Psychol.*, 41, 403–404. doi: 10.1037/h0044670

Braun, J. R. (1962b). Differential susceptibility to faking of various Ghiselli Self-Description Inventory Scales. *Psychol. Rep.*, 10, 639–641. doi: 10.2466/pr0.1962.10.3.639

Braun, J. R. (1963a). Effects of positive and negative faking sets on the Survey of Interpersonal Values. *Psychol. Rep.*, 13, 171–173. doi:10.2466/pr0.1963.13.1.171

Braun, J. R. (1963b). Fakability of the Gordon Personal Inventory: Replication and extension. *J. Psychol.,* 55, 441–444. doi: 10.1080/00223980.1963.9916638

Braun, J. R. (1965a). Effects of specific instructions to fake on Gordon Personal Profile scores. *Psychol. Rep.,* 17, 847–850. doi: 10.2466/pr0.1965.17.3.847

Braun, J. R. (1965b). Note on a faking study with the Myers-Briggs Type Indicator. *Psychol. Rep.*, 17, 924–924. doi: 10.2466/pr0.1965.17.3.924

Braun, J. R., and Alexander, S. (1961). Fakability of the Short Forced-Choice Anxiety Scale. *Psychol. Rep.*, 9, 118. doi: 10.2466/pr0.1961.9.1.118

Braun, J. R., and Farrell, R. M. (1974). Re-examination of the fakability of the Gordon Personal Inventory and Profile: A reply to Schwab. *Psychol. Rep.,* 34, 247–250. doi: 10.2466/pr0.1974.34.1.247

Braun, J. R., and LaFaro, D. (1967). Effects of a good impression set on the Thorndike Dimensions of Temperament. *J. Educ. Meas.*, 4, 237–240. https://www.jstor.org/stable/1434138

Davis, M. (2006). *The relation between optimism and job performance: An applied setting.* [Master’s thesis]. Florida, O: University of Central Florida.

Dicken, C. F. (1959). Simulated patterns on the Edwards Personal Preference Schedule. *J. Appl. Psychol.*, 43, 372–378. doi: 10.1037/h0044779

Dunnette, M. D., McCartney, J., Carlson, H. C., and Kirchner, W. K. (1962). A study of faking behavior on a forced‐choice self‐description checklist. *Pers. Psychol.*, 15, 13–24. doi: 10.1111/j.1744-6570.1962.tb01843.x

Fineman, S. (1975). The Work Preference Inventory: A measure of managerial need for achievement. *J. Occup. Psychol.*, 48, 11–32. doi: 10.1111/j.2044-8325.1975.tb00293.x

Fluckinger, C. D. (2010). *Measurement of Big Five Personality via Q-Sort: comparison with a likert measure and test-taker perceptions and reactions* [Doctoral dissertation]. Akron, OH: University of Akron.

Ford, K. A., and Gunderson, E. K. (1962). *Personality characteristics (EPPS) of antarctic volunteers (Nº NMNRU-62-18)*. San Diego, CA: Navy Medical Neuropsychiatric Reseacrh Unit.

Furnham, A., and Craig, S. (1987). Fakeability and correlates of the perception and preference inventory. *Pers. Individ. Differ*., 8, 459–470. doi: 10.1016/0191-8869(87)90207-8

Gordon, L. V., and Stapleton, E. S. (1956). Fakability of a forced-choice personality test under realistic high school employment conditions. *J. Appl. Psychol.*, 40, 258–262. doi: 10.1037/h0043595

Guan, L. (2015). *Personality, faking, and the ability of identify criteria: Can forced choice formats untangle their relationships?* [Master’s thesis]. Charlottesville, VA: University of Virginia.

Hedberg, R. (1962). More on forced-choice test fakability. *J. Appl. Psychol.*, 46, 125–127. doi: 10.1037/h0038453

Helton, K. T., and Street Jr, D. R. (1992). *The five-factor personality model and naval aviation candidates (Nº NAMRL-1379)*. Pensacola, FL: Naval Aerospace Medical Research Lab.

Hirsh, J. B., and Peterson, J. B. (2008). Predicting creativity and academic success with a “fake-proof” measure of the Big Five. *J. Res. Pers.*, 42, 1323–1333. doi: 10.1016/j.jrp.2008.04.006

Kaess, W. A., and Witryol, S. L. (1957). Positive and negative faking on a forced-choice authoritarian scale. *J. Appl. Psychol.*, 41, 333–339. doi: 10.1037/h0043451

Kirchner, W. K. (1962). " Real-life" faking on the Edwards Personal Preference Schedule by sales applicants. *J. Appl. Psychol.*, 46, 128–130. doi: 10.1037/h0039528

Kirchner, W. K., Dunnette, M. D., and Mousley, N. (1960). Use of the Edwards Personal Preference Schedule in the selection of salesmen. *Pers. Psychol.*, 13, 421–424. doi: 10.1111/j.1744-6570.1960.tb02099.x

Krug, R. E. (1958). The effect of specific selection sets on a forced-choice self-description inventory. *J. Appl. Psychol.*, 42, 89–92. doi: 10.1037/h0044420

Larson, N. L., Lewis, R. J., O’Neill, T. A., and Carswell, J. J. (2013, April). *Are forced choice personality measures contaminated by general mental ability?* [Poster presentation]. 28th Annual Conference of the Society for Industrial and Organizational Psychology, Houston, TX, United States.

Mahar, D., Coburn, B., Griffin, N., Hemeter, F., Potappel, C., Turton, M., and Mulgrew, K. (2006). Stereotyping as a response strategy when faking personality inventories. *Pers. Individ. Differ.*, 40, 1375–1386. doi: 10.1016/j.paid.2005.11.018

Mahar, D., Cologon, J., and Duck, J. (1995). Response strategies when faking personality inventories in a vocational selection setting. *Pers. Individ. Differ.*, 18, 605–609. doi: 10.1016/0191-8869(94)00200-C

Martin, N. R., and Theys, E. (2019). *Development and validation of Aon Hewitt’s personality model and Adaptive Employee Personality Test (ADEPT-15)* [Technical report].

Mourer, S. A. (1959). *A statistical analysis of the relations between interpersonal perception and adjustment in leaders* [Master’s thesis]. Lincoln, NE: University of Nebraska.

Norman, W.T. (1963b). Personality measurement, faking, and detection: An assessment method for use in personnel selection. *J. Appl. Psychol.*, 47, 225–241. doi: 10.1037/h0042106

O'Neill, T. A., Lewis, R. J., Law, S. J., Larson, N., Hancock, S., Radan, J., Lee, N., and Carswell, J. J. (2017). Forced-choice pre-employment personality assessment: Construct validity and resistance to faking. *Pers. Indivi. Differ.*, 115, 120–127. doi: 10.1016/j.paid.2016.03.075

Pavlov, G., Maydeu-Olivares, A., and Fairchild, A. J. (2019). Effects of applicant faking on Forced-Choice and Likert scores. *Organ. Res. Methods.*, 22, 710–739. doi: 10.1177/1094428117753683

Rusmore, J. T. (1956). Fakability of the Gordon Personal Profile. *J. Appl. Psychol*., 40, 175–177. doi: 10.1037/h0042524

Schwab, D. P. (1971). Issues in response distortion studies of personality inventories: A critique and replicated study. *Pers. Psychol.*, 24, 637–647. doi: 10.1111/j.1744-6570.1971.tb00377.x

Sheppard, C., Bates, C., Fracchia, J., and Merlis, S. (1974). Psychological need structures of law enforcement officers. *Psychol. Rep.*, 35, 583–586. doi: 10.2466/pr0.1974.35.1.583

Spector, A. J. (1957). The Attitudes Test in Human Relations (ATHURE). *J. Appl. Psychol.*, 41, 209–213. doi: 10.1037/h0048266

Stollak, G. E. (1965). EPPS performance under social desirability instructions: college females. *Psychol. Rep.*, 16, 119–122. doi: 10.1037/h0022382

Underhill, C. M., Bearden, R. M., & Chen, H. T. (2008). *Evaluation of the fake resistance of a forced-choice paired-comparison computer adaptive personality measure (Nº NPRST-TR-08-2)*. Millington, TN: Navy Personnel Research Studies and Technology.

Vasilopoulos, N. L., Cucina, J. M., Dyomina, N. V., Morewitz, C. L., and Reilly, R. R. (2006). Forced-choice personality tests: A measure of personality and cognitive ability? *Hum. Perform.*, 19, 175–199. doi: 10.1207/s15327043hup1903_1
